# Comparative Mapping Combined With Map-Based Cloning of the *Brassica juncea* Genome Reveals a Candidate Gene for Multilocular Rapeseed

**DOI:** 10.3389/fpls.2018.01744

**Published:** 2018-11-27

**Authors:** Cuiping Chen, Lu Xiao, Xin Li, Dezhi Du

**Affiliations:** State Key Laboratory of Plateau Ecology and Agriculture of Qinghai University, Key Laboratory of Spring Rape Genetic Improvement of Qinghai Province, National Key Laboratory Breeding Base for Innovation and Utilization of Plateau Crop Germplasm, Academy of Agricultural and Forestry Sciences of Qinghai University, Xining, China

**Keywords:** *Brassica juncea*, multilocular, fine mapping, map-based cloning, *CLV1*

## Abstract

Multilocular traits exist in a variety of plants and exert important effects on plant yield. Previous genetic studies have shown that multilocular trait of the *Brassica juncea* cultivar Duoshi is controlled by two recessive genes, *Bjln1* and *Bjln2*. In previous studies, the *Bjln1* gene is located on chromosome A07, and the *Bjln1* candidate gene is *BjuA07.CLV1*. In this study, a BC4 mapping population for the *Bjln2* gene was generated. This population was used to construct genetic linkage maps of the *Bjln2* gene using amplified fragment length polymorphism (AFLP), intron length polymorphism (IP) and simple sequence repeat (SSR) methodology. The results showed that the *Bjln2* gene was restricted to a 0.63 cM interval. BLAST alignment with *B. juncea* revealed the *Bjln2* gene was located within a 11.81–16.65 Mb region on chromosome B07. Moreover, the candidate gene *BjuB07.CLV1* (equivalent to *Bjln2*) was cloned by comparing mapping and map-based cloning, and *BjuB07.CLV1* gene was shown to have the ability to restore the bilocular traits in a genetic complementation experiment. The sequencing revealed that a 4961 bp insertion interrupted the coding sequence of the *BjuB07.CLV1* gene, resulting in an increase in locule number. Expression analysis revealed that *BjuB07.CLV1* was expressed in all tissues and the expression level in bilocular plants was significantly higher than that in multilocular plants. In addition, markers closely linked to the *Bjln2* gene were developed and used for molecular marker-assisted breeding of multilocular traits.

## Introduction

Rapeseed is among the most important oil crops. Most rapeseed plants produce siliques with two locules, these plants are referred to as bilocular rapeseed. However, a small number of varieties develop siliques with three or four locules, these varieties are referred to as multilocular rapeseed. Previous studies have shown that the yield per plant of multilocular rapeseed is significantly greater than that of bilocular rapeseed in the same genetic background (Zhao et al., 2003a). The three most important components of rapeseed yield are the number of siliques per plant, the number of seeds per silique, and seed weight. Previous studies have shown that the increase in yield per multilocular rapeseed plant were mainly from the greater number of seeds per silique. Therefore, the study of multilocular traits is highly important meaning for improving rapeseed yield (Katiyar et al., 1998; Zhao et al., 2003a,b).

Rapeseed siliques develop from gynoecia. The pistil usually comprises two carpels that grow and combine with each other to generate a locule (or ovary). As the ovary develops, the medial ridges grow toward each other and form a false septum. The ovary is then divided into two locules by the septum. However, multilocular rapeseed produce siliques with two or more false septum, thus dividing the ovary into multiple locules. Two pseudosepta grow in parallel in the ovaries of the trilocular *B. juncea* line J163-4, dividing the ovary into three locules (Xu et al., 2014). The two parallel pseudosepta resemble a “II” shape, and the middle chamber is clearly larger than the others (Xu et al., 2014). *Brassica rapa* “Yellow Sarson” develops a tetralocular ovary that contains four septas (Yadava et al., 2014).

A number of studies have shown that multilocular traits of *B. rapa* and *B. juncea* can be stably inherited and controlled by one or two recessive genes. The gene that controls the multicarpel phenotype in *B. rapa* was mapped onto chromosome A4. Sequence comparison revealed that a C → T transition in *Bra034340* caused the multicarpel phenotype in *B. rapa* (Fan et al., 2014; Yadava et al., 2014). In *B. juncea*, the trilocular gene *Bjmc1* is located within the B genome, and insertion of a copia long terminal repeat (LTR) retrotransposable element in an exon gives rise to the trilocular phenotype (Xu et al., 2014, 2017). The multilocular gene *Bjln1*, which is derived from the *B. juncea* cultivar Duoshi, was delimited to an 85 kb region within linkage group A07 (Xiao et al., 2018).

There is a relationship between the shoot apical meristem (SAM) and the formation of multilocular traits. Specifically, the formation of multilocular phenotype is related to the enlargement of the SAM (Clark et al., 1997). Molecular genetic studies have shown that the *WUSCHEL* (*WUS*) gene encodes a homologous-domain transcription factor, that maintains the number of stem cells and inhibits stem cell differentiation (Mayer et al., 1998). In contrast, *CLAVATA* (*CLV*) genes (*CLV1* and *CLV3*) promote the differentiation of stem cells and the formation of organs (Clark et al., 1996). *WUS* and *CLV* form a feedback loop that regulates the balance between the number of stem cells and differentiation of progenitor cells (Xie et al., 2009). Mutations in the *CLV* gene (including *CLV1, CLV2*, and *CLV3*) of *Arabidopsis* led to an abnormal increase in the number of stem cells, giving rise to the enlargement of flower organs (Clark et al., 1997; Brand et al., 2000; Schoof et al., 2000). In addition, Hu et al. (2018) recently reported a new group of receptor kinases, *CLAVATA 3 INSENSITIVE RECEPTOR KINASEs* (*CIKs*), that can sense the expression of *WUS* within the *CLV3* signal to maintain the homeostasis of the SAM. Despite this progress, the molecular and genetic mechanisms underlying multilocular traits are still poorly understood.

Previous genetic studies have shown that multilocular trait of Duoshi is controlled by a pair of recessive nuclear genes, *Bjln1* and *Bjln2* (Xiao et al., 2013). The *Bjln1* gene is located on chromosome A07 of *B. juncea*, and the candidate gene of *Bjln1* is *BjuA07.CLV1* (Xiao et al., 2018). The objective of the present study is to map and clone the other multilocular gene (*Bjln2*) by comparing mapping results with map-based cloning, and to understand the function of the *Bjln2* gene.

## Materials and methods

### Plant materials and population construction

The BC4 population (MB2013) consisting of 959 individuals was derived from a cross between Duoshi (multilocular cultivar) and Tayou2 (bilocular cultivar). Tayou2 is a normal *B*. *juncea* cultivar and produces siliques with 2 locules, whereas the landrace Duoshi, which originated from the Qinghai-Tibetan plateau, produces siliques with 3–4 locules. One individual F1 plant was choiced and backcrossed with Duoshi to generate a BC1 population. The bilocular plants (bl) randomly selected from the BC1 were continuously backcrossed with multilocular plants (ml) to produce a BC2 population. The resulting BC2 population was used to examine the locules of siliques. Each BC2 population displaying a 1:1 (ml:bl) ratio was subsequently used for allelism analysis in conjunction with a BC3 population (hereafter referred to as MB2011) that was used for *Bjln1* mapping in a previous study (Xiao et al., 2013). Molecular markers that were tightly linked to *Bjln1* were used to screen the BC2 lines. In some BC2 lines, there was no linkage between the tested markers and the number of locules in the siliques, suggesting that these BC2 lines were not allelic to MB2011. These lines were subsequently backcrossed with Duoshi for two generations. The resulting BC4 segregating line (hereafter referred to as MB2013) comprising 959 plants was used to map and clone the *BjLn2* gene. The bilocular plants with the genotype *Bjln1ln1Ln2ln2* produced a typical silique that contained two locules, whereas the multilocular plants with the genotype *Bjln1ln1ln2ln2* produced siliques that contained 3–4 locules. The locule number trait was investigated visually. Plants having more than 90% multilocular siliques per plants were classified as multilocular plants, whereas plants with no multilocular siliques were considered as bilocular plants.

A single bilocular individual within the MB2013 population was randomly selected and self-pollinated. Sixteen bilocular plants were randomly selected from the resulting BC4F2 population and self-pollinated. Each BC4F3 population comprising only bilocular individuals was successively maintained by self-pollination for two generations, thus producing BC4F5 plants homozygous for *BjLn2*. The multilocular plants in the BC4F2 population were self-pollinated for 3 generations, and individual BC4F5 plants homozygous for *Bjln2* were obtained. The homozygous bilocular and multilocular plants in the BC4F5 population were NILs and used for gene cloning, histological analysis and quantitative real-time PCR (qRT-PCR) analysis.

The transgenic plants were grown in an illuminated incubator that had a 16:8 h light:dark photoperiod at 24 and 16°C, respectively. The *Arabidopsis* mutant *CS45* and wild type *Columbia* was purchased from the Arabidopsis Biological Resource Center (ABRC) mutant collection (https://abrc.osu.edu//).

### Allelism analysis

**Figure 1:**
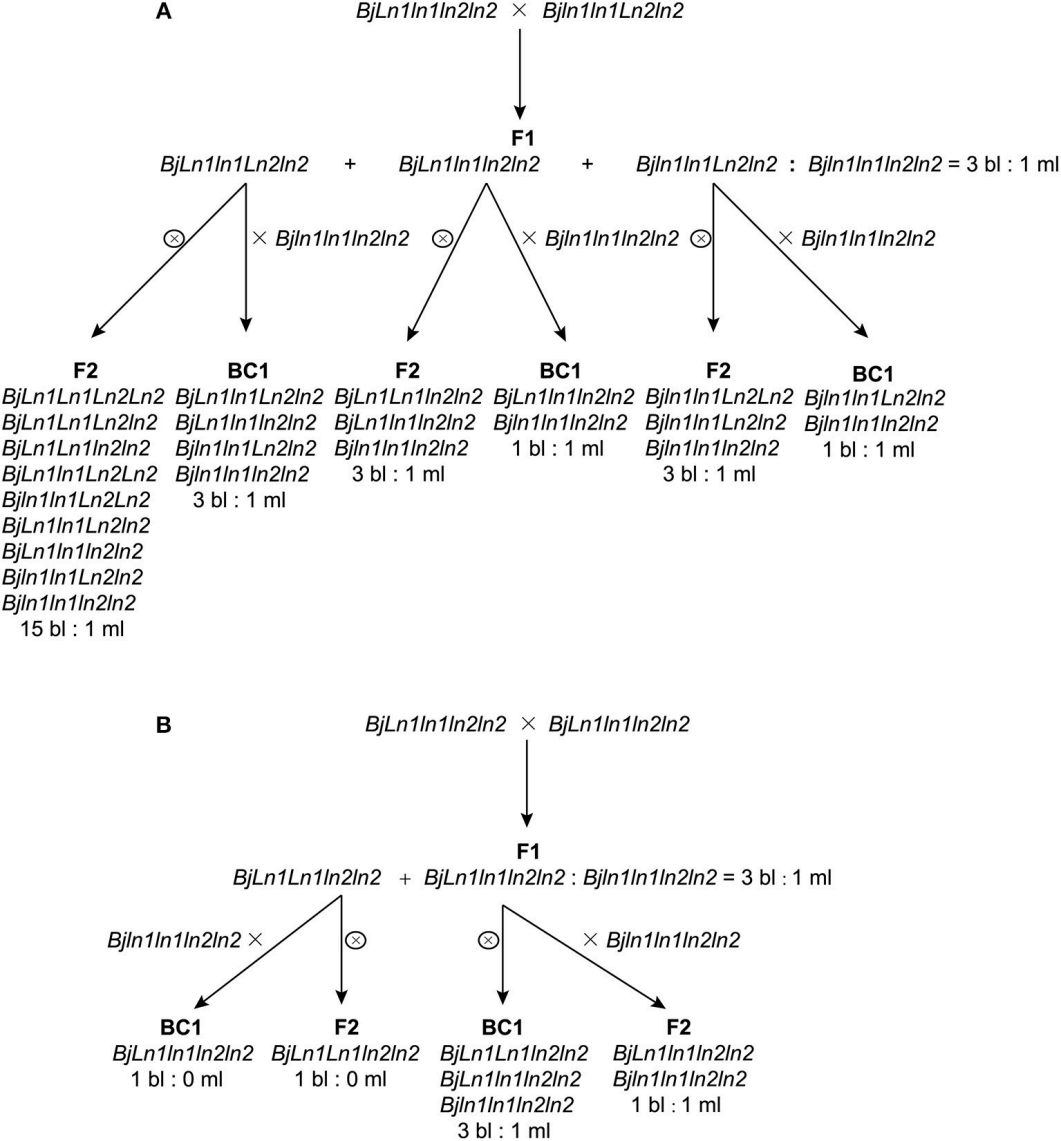
Allelism analysis. **(A)** Supposed segregation performance in the population derived from the cross population of bilocular plants of MB2011 and MB2013, in which the genotype of bilocular plants of MB2011 is *BjLn1ln1ln2ln2* and the genotype of bilocular plants of MB2013 is *Bjln1ln1Ln2ln2*. The results show that bilocular plants of MB2011 are not allelic to bilocular plants of MB2013. **(B)** Supposed segregation performance in the population derived from the same cross, in which the genotype of bilocular plants of MB2011 is *BjLn1ln1ln2ln2* and the genotype of MB2013 is *BjLn1ln1ln2ln2*. The results show that bilocular plants of MB2011 are allelic to MB2013. bl represents bilocular plants; ml represents multilocular plants.

MB2013 bilocular plants (expected genotype: *Bjln1ln1Ln2ln2*) were crossed with MB2011 bilocular plants (genotype: *BjLn1ln1ln2ln2*) for allelism analysis. The randomly selected F1 bilocular plants were subsequently backcrossed with Duoshi, generating a BC1 generation (Table 1). These bilocular plants were self-pollinated to obtain an F2 population (Table 2). The allelic relationship between MB2013 and MB2011 was determined based on the segregation ratio of the bilocular and multilocular plants in the resulting population. If MB2013 was not allelic to MB2011, there would be a segregation ratio of 3:1 (ml:bl) in addition to a ratio of 1:1 (ml:bl) in the BC1 generation. For the F2 population, there would be a 15:1 (ml:bl) segregation ratio expected in addition to the 3:1 (ml:bl) ratio. The detailed methodology of the allelism analysis is shown in Figure 1A. However, if MB2013 was allelic to MB2011, two types of populations in the resulting BC1 population would exist: one composed of all bilocular plants and another exhibiting a segregation ratio of 1:1 (ml:bl). In the case of the F2 generation, two types of populations would exist: one consisting of all bilocular plants and another exhibiting a 3:1 (ml:bl) segregation ratio (Figure 1B).

**Table 1:** Segregation analyses of locule number in BC1 generation.

**Line**	**No. of tested plants**			**Expected ratio**	**χ^2^**
	**Bilocular**	**Multilocular**	**Total**	
15A-148 to 15A-151	19	15	34	1:1	0.4706
15A-152 to 15A-155	42	11	53	3:1	0.5094
15A-156 to 15A-159	28	23	51	1:1	0.4902
15A-160 to 15A-163	24	20	44	1:1	0.3636
15A-164 to 15A-167	56	10	66	3:1	3.4141
15A-168 to 15A-171	27	30	57	1:1	0.1579

*$\chi_{0.05,1}^{2}$ = 3.84*.

**Table 2:** Segregation analyses of locule number in F2 generation.

**Line**	**No. of tested plants**			**Expected ratio**	**χ^2^**
	**Bilocular**	**Multilocular**	**Total**	
15A-100 to 15A-107	91	35	126	3:1	0.5185
15A-108 to 15A-115	112	8	120	15:1	0.0356
15A-116 to 15A-123	102	8	110	15:1	0.1964
15A-124 to 15A-131	95	7	102	15:1	0.1026
15A-132 to 15A-139	80	26	106	3:1	0.0126
15A-140 to 15A-147	76	21	97	3:1	0.5808

*$\chi_{0.05,1}^{2}$ = 3.84*.

### AFLP, SSR, and indel marker development

The genomic DNA of the MB2013 bilocular plants and multilocular plants was isolated via a modified cetyl-trimethylammonium bromide (CTAB) method (Doyle et al., 1990). Two bilocular bulks and two multilocular bulks were constructed for molecular marker identification by pooling equal amounts of DNA from 6 bilocular plants and 6 multilocular plants, respectively.

Amplified fragment length polymorphism (AFLP) was first used to screen markers that were tightly linked to *Bjln2* in accordance with the protocol developed by Vos et al. (1995) with slight modifications (Lu et al., 2004; Lei et al., 2007). The selected restriction enzyme combinations were *Sac*I/*Mse*I and *EcoR*I/*Mse*I. The restricted DNA was subsequently ligated to specific double-stranded adaptors and then screened with the pre-amplified primer combinations (SA/MC, SA/MG, and EA/MC). The pre-amplified primers are composed of 17 bases. The pre-amplification product was then diluted (1:30) and used for selective amplification. The selective amplification primers are added with 3 selective bases on the basis of the pre-amplified primers. The products of the selective amplification and an equal volume of loading buffer [98% formamide, 0.025% xylene cyanol, 0.025% bromophenol blue, 10 mM EDTA (pH 8.0)] were subsequently mixed together. Denaturing polyacrylamide gel (6%) was used for mixture separation.

To assign the *Bjln2* gene to a specific chromosome, PCR-based primers from publicly available resources, consisting of databases (http://brassicadb.org/brad/, http://www.brassica.info/) and reference maps of *B*. *juncea* and *B*. *carinata* (Ramchiary et al., 2007; Panjabi et al., 2008; Guo et al., 2012; Yadava et al., 2012), were used for marker screening.

After the syntenic interval around *Bjln2* on chromosome B07 of *B*. *juncea var. tumida* was identified, SSR primers were used in accordance the sequence information of the homologous region. Microsatellite screening was then performed with SSR Hunter 1.3 (Li and Wan, 2005), and SSR primers were designed via Primer Premier 5 software. In addition, Indel primers that target within the collinear interval were developed as part of a previous study (Xiao et al., 2018) and applied for polymorphism screening. SSR and Indel amplification were performed as described by Lowe et al. (2002) and Xie et al. (2012), respectively.

### Sequencing of specific polymorphic fragments

The specific bands from the AFLP, SSR and Indel markers were gel purified, re-amplified and then ligated into a pMD18-T vector (Takara, Dalian, China) as described by Xia et al. (2012). The recombinants were cloned into *E*. *coli DH5a*. Five positive clones were sequenced (Shanghai Sangon Biotechnology Corporation, Shanghai, China).

To test the putative collinear interval surrounding *Bjln2* in *B*. *juncea, Brassica nigra* and *B*. *rapa*, sequence alignments were performed using the BLAST search program of the NCBI database (http://www.ncbi.nlm.nih.gov) and the BRAD (http://brassicadb.org/brad/).

### Mapping

A total of 192 individuals from the MB2013 population were used to examine the genetic distances of the 10 AFLP markers (Table 3). After analysis the relative positions of the AFLP markers, the target gene were determined, and two farthest apart markers on both sides of *Bjln2* were used to screen recombinants from the 192 plants. These recombinants were used to test the genetic distance between the newly developed SSR and Indel markers. For further fine mapping, two PCR-based markers, IL37 and EM1, were used to screen the remaining individuals of the MB2013 population for recombinant identification. All co-segregated markers identified in the first round were further used to analyze these recombinants (Table 4).

**Table 3:** AFLP primers used in mapping of the *Bjln2* gene.

**Primer name**	**Sequence 5^′^-3^′^**	**Fragment size (bp)**
af01 (SA09MG10)	GACTGCGTACAAGCTCAACA	184
	GATGAGTCCTGAGTAAGGCT
af02 (SA14MC13)	GACTGCGTACAAGCTCAAGT	178
	GATGAGTCCTGAGTAACCGA
af03 (SA16MC15)	GACTGCGTACAAGCTCAAGG	180
	GATGAGTCCTGAGTAACCGC
af04 (SA09MG01)	GACTGCGTACAAGCTCAACA	220
	GATGAGTCCTGAGTAAGGAA
af05 (SA03MC16)	GACTGCGTACAAGCTCAAAC	485
	GATGAGTCCTGAGTAACCGG
af06 (SA10MC16)	GACTGCGTACAAGCTCAACT	402
	GATGAGTCCTGAGTAACCGG
af07 (SA01MC05)	GACTGCGTACAAGCTCAAAA	169
	GATGAGTCCTGAGTAACCTA
af08 (SA02MC16)	GACTGCGTACAAGCTCAAAT	152
	GATGAGTCCTGAGTAACCGG
af09 (EA13MC04)	GACTGCGTACCAATTCAAGA	556
	GATGAGTCCTGAGTAACCAG
af10 (SA09MG08)	GACTGCGTACAAGCTCAACA	233
	GATGAGTCCTGAGTAAGGTG

**Table 4:** SSR, SCAR, and Indel primers used in mapping of the *Bjln2* gene.

**Primer name**	**Sequence 5^′^-3^′^**	**Fragment size (bp)**
11-46	TATGGGATTTTGCTGCTGAC	171
	AATTGGGTCTTTGATGCGAA
11-52	AACTCATCATCTCTAGGTGA	223
	AGCTCATCATCTCTAGGTGA
13-6-4	CATGCCTACCTTCAGTTTCA	237
	CTCGGAGGATTATCGGTCTA
8-221	TTACCGCCCCTTAGATGATT	225
	TCACCGAAAACAACTCACTC
14-160	TTCAAGTCCAAATCAAACGC	250
	GAGGTAGCAACAGAAAGGAA
EM1	CTTATCTCCCAAGGCAAGTT	181
	AGTGCATCCTTTCATACTCTC
IL37	AGTACCGGTCAAATTGAAAC	137
	TCAATTCAAAGGTCTCCTTC
Ni4C02	TCCCTTGTCTACTTGCGACC	269
	ACCCTTGTTCCCTCATCTCC
SR03	TAACGCTGGAGGGACATACTTT	186
	GGTTGTTACCCCGAAGATGATA
SR07	CCAGGTTAGAGAAGTGAGAG	208
	GGATAGACTCAGAGATGCCT
SC09	CTTGTTCCTCCTCGAAGCACTGA	506
	ACCGCTGTTGCACTTGCTCTAAC

### Cloning of the candidate gene

The homozygous bilocular and multilocular plants in the BC4F5 population were used to amplify the full-length gDNA and cDNA of the target gene. Specific primers (P1, P2, and P3) were designed according to the sequence of *Bra015812*, the homologous gene of *BjLn2* in *B*. *rapa*. After the fragments were recovered and compared sequencing with the *B. juncea*, Cd1, Cd2, Cd3, and Cd4 primers were designed and amplified the fragment of *BjLn2* gene. The gDNA and cDNA of the bilocular materials were cloned by G1-F/G6-R and Cd1F/Cd4R, respectively (Table 5). The cDNA of the multilocular material was cloned via primer Cd1F/Cd3R combined a 3′-Full RACE Core Set Version 2.0 (TaKaRa, Dalian, China). Comparative analysis of the candidate genes among the bilocular and multilocular sequences was conducted by Lasergene.

**Table 5:** Primers used in gene cloning and expression.

**Primer name**	**Sequence 5^′^-3^′^**	**Purpose**
G1-F	TATGACCATGATTACGAATTCGTGTGTGGCCATTGATAGA	Cloning the *BjLn2* Gene
G6-R	GCCAAGCTTGCATGCCTGCAGTGGATTCGTCGTTGTAGTTT
Cd1	AAAACTCACGCTAACTTCTT	Cloning the *BjLn2* Gene
	CCATGTCGAGGATTTCTA
Cd2	TTAACGAAGCTAGAAATCC	Cloning the *BjLn2* Gene
	TCGCGAGAATGAGTCAGG
Cd3	CACGAAGATCAACACGAG	Cloning the *BjLn2* Gene
	AGATGACCGCCTTTAGAC
Cd4	TCTTCAGTGGGAGACGAG	Cloning the *BjLn2* Gene
	ATTAGCCTTTGATTGGGT
P1	CTCCAGATCATCATCATCAT	Cloning the *BjLn2* Gene
	TCTTCAACGACACTTCCTTC
P2	GTTAGCTTCCAGGAGAGAGA	Cloning the *BjLn2* Gene
	CCGCTAGAGATGAAGAGTCT
P3	GTGAAGTTGTTGTTGTACGC	Cloning the *BjLn2* Gene
	CACAGCAGAAGAAGGTCTAC
M13F	CAGGAAACAGCTATGAC	Identifying the transgenic plants
C-2F	TGTTTGTCTGCTCTTGTTGA	Identifying the transgenic plants
M13R	CCCAGTCACGACGTTGTAAAACG
A-Actin2	AAGATCTGGCATCCACTTTC	qPCR analysis
	TAGTCAACAGCAACAAAGGAG
CLV-2	ACGGTTAGTTGGACGCGGAA	qPCR analysis
	CCCACTGAAGATGACCGCCT

### Genetic complementation experiment of the *Bjln2* gene

For complementation tests, a 6,373 bp genomic fragment that contained the *BjLn2* gene as well as 2,237 bp upstream, 3,154 bp coding regions and 983 bp downstream of the transcribed region, was amplified from the genomic DNA of the bilocular plants via G1-F/G6-R primers (Table 5). The correct fragment was confirmed by sequencing and cloned into the *EcoR*I-*Pst*I sites of a pCAMBIA2300 vector using a One Step Cloning Kit (Vazyme, Nanjing, China) to produce *pBjuB.CLV1*:*BjuB.CLV1*. After the fused constructs were verified, the construct was transformed into *Arabidopsis CS45* mutants via the *A. thaliana* inflorescence infiltration method. The *pBjuB.CLV1*:*BjuB.CLV1* construct was transformed into multilocular plants via *Agrobacterium-*mediated transformation simultaneously. The positive transgenic plants were screened and confirmed by antibiotic selection and PCR detection with M13F/Cd4R and C-2F/M13R primers (Table 5).

### RNA extraction and quantitative real-time PCR

The total RNA was extracted from different tissues of the homozygous bilocular and multilocular plants using a TaKaRa Mini BEST Plant RNA Extraction Kit (TaKaRa, Dalian, China). The materials included the roots and leaves at the seedling stage; the stems, SAMs and buds at the budding stage; the flowers, pistils and stem leaves at the flowering stage; and the siliques at the mature stage. Take three replicate samples per tissue. The concentration of the RNA samples was adjusted to 500 ng/μL using Biophotometer Plus (Expander, Germany). The cDNA was reverse transcribed via a Prime Script^TM^ RT reagent kit with gDNA Eraser (Perfect Real Time) (TaKaRa, Dalian, China) and then diluted to 12.5 ng/μL. The PCR primers were designed with Primer Premier 5.0 (Premier Biosoft International, Palo Alto, CA, USA). A-Actin2 was used as a reference gene for the relative quantification of transcript levels (Muthukumar et al., 2007). The CLV-2 primer was designed in accordance with the *CLV1* gene of *B. juncea* (Table 5). Primers were also diluted to 12.5 ng/μL. qRT-PCR (8 μL cDNA samples, 1 μL left primer and 1 μL right primer) was performed using SYBR^®;^ Premix Ex Taq^TM^ (Tli RNaseH Plus) (TaKaRa, Dalian, China) on a LightCycler^®;^ 480 Instrument II (Roche, Switzerland). The 2^−ΔΔ*Ct*^ values for the gene were analyzed using Microsoft Excel and used to detect the expression level between bilocular and multilocular plants. Each expression profile was independently verified for three biological replicates, and each biological replicate do two technical replicates.

### Paraffin sections

To observe the phenotypic differences between the homozygous bilocular and multilocular materials among the NILs, the SAMs and flower buds were separated under a dissecting microscope, fixed in 50% formalin-acetic acid-alcohol (FAA) solution for 24 h, and then transferred to safranin dye for 5 d. The tissues were then successively placed in 30% ethanol, 50% ethanol, 75% ethanol, and 100% ethanol for 2 h each. After they were made transparent, waxed, embedded, sliced and sealed, the tissues were imaged under a microscope. The surface area of the SAM was defined by measuring the distance of the meristem dome above a straight line between the top edges of the smallest leaf primordia. The mean value was calculated from the measurements of more than 3 SAMs for each NIL.

## Results

### Analysis of MB2013 and MB2011 allelism

One bilocular MB2013 (expected genotype: *Bjln1ln1Ln2ln2*) was randomly selected and crossed with bilocular MB2011 individuals (genotype: *BjLn1ln1ln2ln2*). The obtained F1 exhibited a 3:1 (bi:ml) separation. Six bilocular (bl) plants of F1 were randomly selected and subsequently backcrossed with the multilocular parent Duoshi to produce a BC1 generation, in which three BC1 plants showed a 3:1 (bl:ml) segregation ratio, whereas another three BC1 plants displayed a 1:1 (bl:ml) segregation ratio (Table 1). The six bilocular F1 plants were simultaneously self-pollinated. Three F2 populations exhibited a 15:1 (bl:ml) segregation ratio, and the remaining three populations exhibited a 3:1 (bl:ml) segregation ratio (Table 2). No population fully comprising bilocular plants was observed among the BC1 and F2 generations. These results demonstrate that the segregating locus in MB2013 was not allelic to that in MB2011, and the genotype of the bilocular plants in MB2013 was *Bjln1ln1Ln2ln2* (Figure 1).

### Identification of AFLP markers and primary linkage analysis

**Figure 2:**
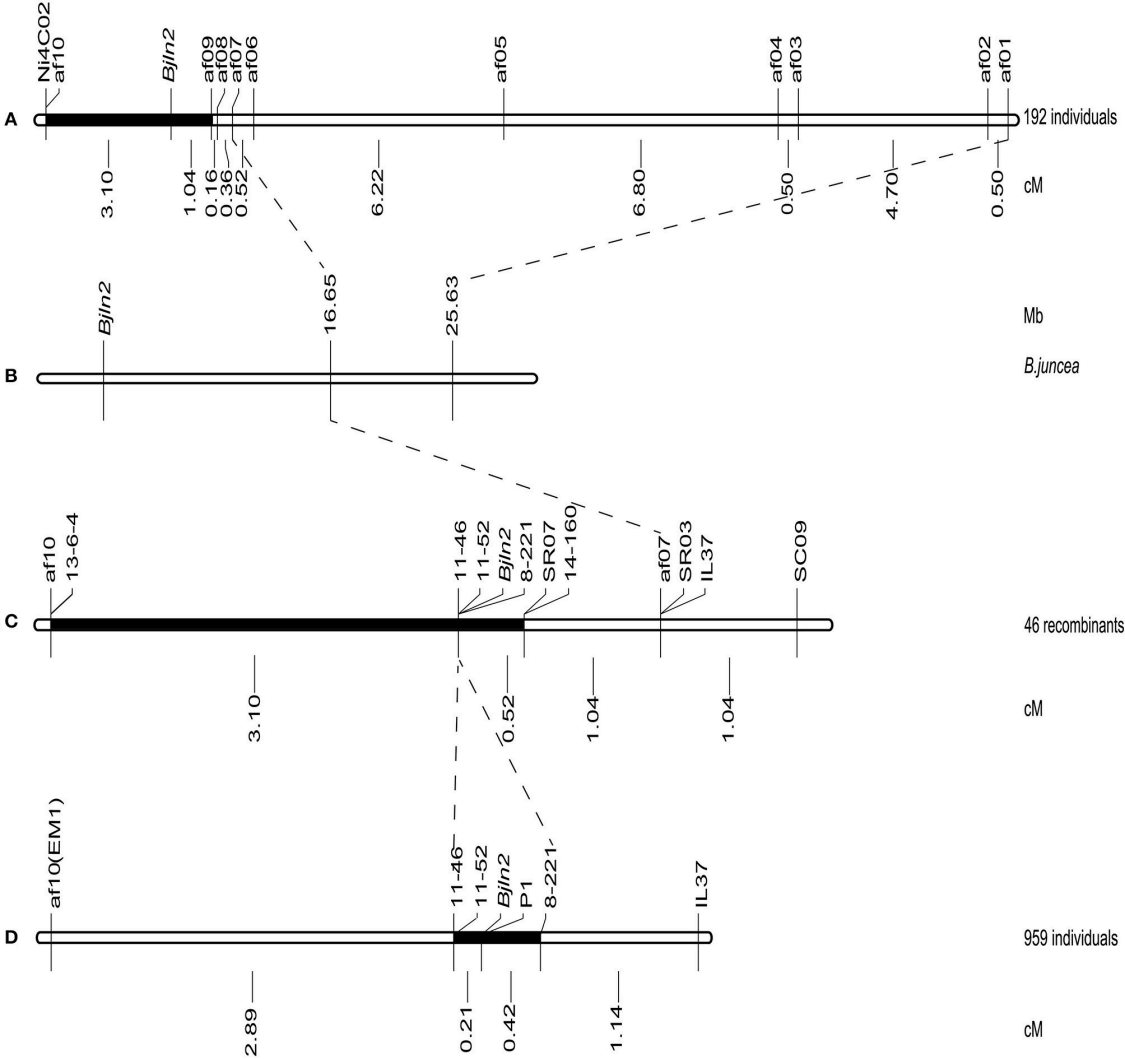
Mapping and fine mapping of the *Bjln2* gene. The black boxes represent the candidate interval of the *Bjln2* gene. The genetic distances are displayed in cM and physical distances are displayed in Mb for the *Bjln2* gene according to the NCBI database. **(A)** The genetic linkage map of the *Bjln2* gene obtained by screening the 192 individuals of MB2013 population. **(B)** The physical position of the *Bjln2* gene on B07 chromosome of *B. juncea*. **(C)** The genetic linkage map of the *Bjln2* gene with 46 recombinant individuals (by screening the 192 individuals with af1 and af10). **(D)** Fine mapping of the *Bjln2* gene with 959 individuals of MB2013.

AFLP combined with bulked segregant analysis (BSA) was applied to screen the putative markers linked to *Bjln2*. A total of 512 AFLP primer combinations were screened, and 10 AFLP primers displayed polymorphisms between multilocular and bilocular bulks. Therefore, those 10 AFLP primers were identified as markers that were tightly linked to the *Bjln2* gene and were designated af01 to af10. Those 10 AFLP markers were subsequently used to screen 192 plants that were randomly selected from the MB2013 population for a preliminary linkage analysis. The results indicated that 9 of the 10 markers were located on one side of the *Bjln2* gene and only 1 of the 10 was on the other side of the *Bjln2* gene. af09, the flanking marker on one side, was located 1.04 cM away from the *Bjln2* gene, whereas af10, the flanking marker on the other side, was 3.10 cM from the target gene (Figure 2A).

### Linkage group assignment and marker identification

To assign the *Bjln2* gene to a specific linkage group, PCR-based markers from the reference maps of *B. juncea* and *Brassica carinata* (Ramchiary et al., 2007; Panjabi et al., 2008; Guo et al., 2012; Yadava et al., 2012) were applied. After screening 158 collected primers, only one anchor mark Ni4C02 (from Guo et al., 2012), was located on chromosome B07 of *B. juncea*, displayed polymorphism between the multilocular and bilocular bulks.

With the release of the genome sequencing of *B. juncea var. tumida*, all polymorphic fragments of the 10 AFLP markers and one SSR marker (Ni4C02) were sequenced and submitted to the National Center for Biotechnology Information (NCBI) database (http://www.ncbi.nlm.nih.gov). The results indicated that only two AFLP markers (af01 and af07) had homologs on chromosome B07 of *B. juncea*. Markers af01 (positioned at 25.63 Mb of B07) and af07 (positioned at 16.65 Mb of B07) were found to have been mapped slightly closer to *Bjln2*. These results suggested that the region positioned at < 16.65 Mb of B07 may harbor the target gene (Figure 2B).

The sequence information surrounding the interval < 16.65 Mb of chromosome B07 was subsequently used to explore the new SSR markers. As a result, a total of 6 SSR markers (14-160, SR07, 8-221, 11-46, 11-52, and 13-6-4) were identified as polymorphic markers. In addition, 48 insertion/deletion (Indel) primers that target within the putative interval of B07 were identified in a previous study, and one Indel marker, IL37, was identified. These results further confirmed that the *Bjln2* gene is located on B07 of *B. juncea*.

Moreover, PCR-based markers used in studies related to the multilocular trait in *Brassica* (Xu et al., 2013; Fan et al., 2014; Wang et al., 2016) were used for polymorphism screening in our mapping population, and 2 markers (SC09 and SR03) were identified.

### Fine mapping of the *Bjln2* gene within a 4.84-Mb interval of *B*. *juncea*

The two farthest markers (af01 and af10) on both sides of *Bjln2* gene were used to analyze the 192 individual plants; 39 and 7 recombinants were obtained, respectively. These 46 recombinants were subsequently used to analyze the 9 newly developed markers (14-160, SR07, 8-221, 11-46, 11-52, 13-6-4, IL37, SC09, and SR03) for genetic distance identification. The results indicated that three markers (8-221, 11-46, and 11-52) co-segregated with the target gene, 5 markers (SC09, IL37, SR03, 14-160, and SR07) were located on the same side as was af01, and one marker (13-6-4) was on the opposite side. The *Bjln2* gene was further delimited to a 3.62 cM interval between markers SR07 and af10 (or 13-6-4) (Figure 2C).

**Figure 3:**
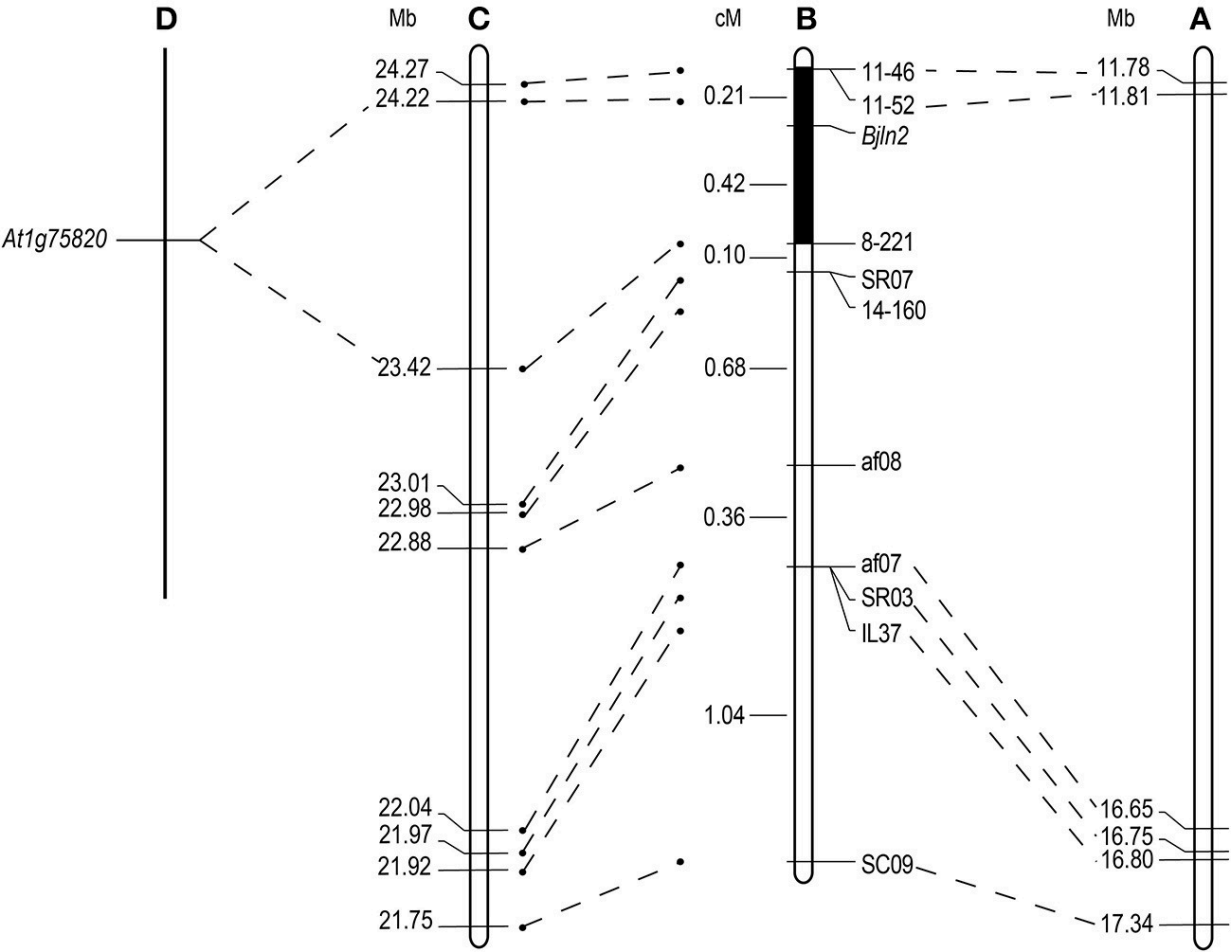
Comparative analysis of the candidate interval for the *Bjln2* gene between *B. juncea, B. rapa* and *Arabidopsis*. Genetic distances are displayed in cM and physical distances are displayed in Mb for the *Bjln2* gene according to the NCBI and BRAD database. **(A)** The physical position of the *Bjln2* gene on B07 chromosome of *B. juncea*. **(B)** The genetic linkage map of the *Bjln2* gene with partial markers. The black box represents the candidate interval for the *Bjln2* gene. **(C)** The homologous region between the candidate interval of the *Bjln2* gene and A07 chromosome of *B.rapa*. **(D)** The orthologous gene with *Arabidopsis* in the region between 23.42 Mb- 24.22 Mb of *B. rapa*.

To facilitate screening the individual plants of the whole MB2013 population, the af10 marker was converted to a sequence-characterized amplified region (SCAR) marker, EM1. Two PCR-based markers (IL37 and EM1) were subsequently used to screen the remaining 767 individual plants of MB2013, and 12 and 24 recombinants were identified between the two PCR-based markers and the target gene, respectively. These 36 recombinants were further used to analyze the three co-segregating markers, 8-221, 11-46 and 11-52. The results indicated that marker 8-221 was located on the same side as af01, whereas markers 11-46 and 11-52 were positioned on the other side of *Bjln2*. Thus, the *Bjln2* gene was restricted to a 0.63 cM interval (Figure 2D). All newly developed SSR and Indel markers were submitted to the NCBI database (http://www.ncbi.nlm.nih.gov) for BLAST analysis. The sequence alignment results suggested that the *Bjln2* gene was positioned within the interval of 11.81–16.65 Mb on B07 of *B. juncea* (Figures 3A,B). However, the sequence information on this region contains considerable gaps.

All AFLP, SSR and Indel markers were used for BLAST analysis against the Brassica Database (BRAD) (http://brassicadb.org/brad/). The analysis revealed that 10 markers (SC09, IL37, SR03, af07, af08, 14-160, SR07, 8-221, 11-52, and 11-46) had homologs on chromosome A07 of *B. rapa*. The sequence alignment results suggested that a syntenic interval on A07 of *B. rapa* exists for the *Bjln2* gene and the surrounding region. On the basis of this information in conjunction with the genetic positions of the 10 markers, we presumed that the region from nucleotides 23.42–24.22 Mb on the bottom of A07 harbors the homologous gene of *Bjln2* (Figure 3C). We then examined this approximately 0.8 Mb interval in which the gene *Bra015812* is orthologous to *At1g75820* in *Arabidopsis* (Figure 3D). *At1g75820* controls the size of the shoot and floral meristem (FM) and contributes to maintaining the identity of the FM. Mutation of *At1g75820* leads to the generation of multiple carpels. Therefore, we speculated that *Bra015812* is homologous to *Bjln2*. On the basis of the sequence information of *Bra015812*, three primers (P1, P2, and P3) were designed to detect polymorphisms between the two bulks. Primer P1, which revealed polymorphism between the bilocular and multilocular plants, was subsequently used to screen the 36 recombinant individuals of the MB2013 population. The results indicated that P1 was co-segregated with the *Bjln2* gene (Figure 2D). The specific fragment of P1 was subsequently sequenced and submitted to the NCBI database (http://www.ncbi.nlm.nih.gov) and the BRAD (http://brassicadb.org/brad/) for sequence alignment.

### Transformation and phenotypic identification of *arabidopsis* mutants

**Figure 4:**
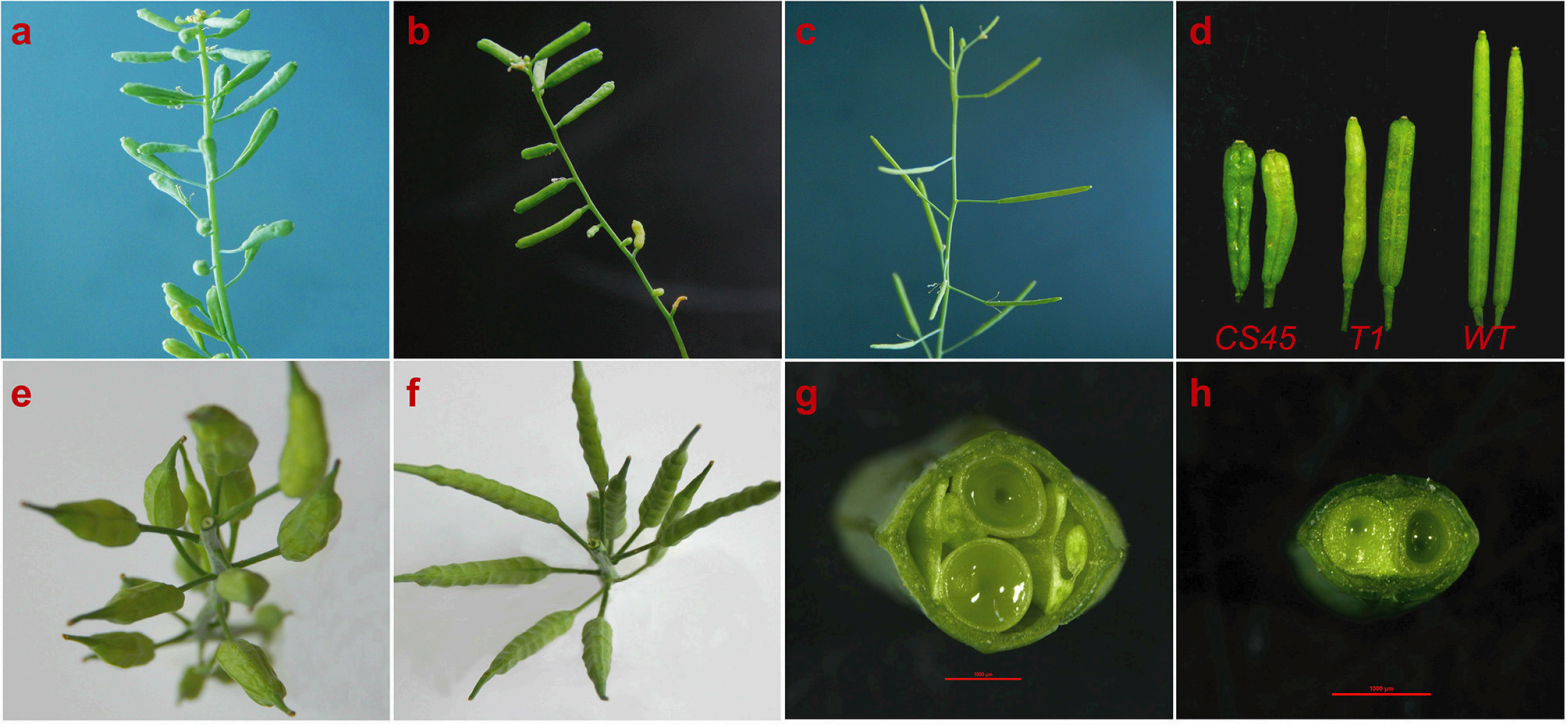
Phenotypes of the complementation experiment. **(a**–**d)** Are the phenotypes of *Arabidopsis* siliques; **(e**–**h)** are the phenotypes of *B.juncea* siliques. **(a)** *CS45*, mutant *Arabidopsis*, with short and four locules per silique. **(b)** T1, transferred the *BjuB.CLV1* into the mutant *CS45*, with two locules per silique in the multilocular background. **(c)** *WT*, with two long locules per silique. **(d)** From left to right, silique phenotypes of *CS45*, T1 and *WT*. **(e)** Phenotype of the Duoshi, non-transgenic plants. **(f)** Generation of two locule-shaped siliques in the *BjuB07.CLV1*/*Bjln2* T_0_ transgenic plant. **(g)** Cross section of the Duoshi silique, non-transgenic plants. **(h)** Cross section of the T_0_ transgenic silique.

To prove that the candidate gene *BjuB07.CLV1* was *BjLn2, pBjuB07.CLV1:BjuB07.CLV1* was constructed and cloned into a modified pCAMBIA2300 vector. A 6,373 bp *BjLn2* fragment was obtained from the bilocular plants of near-isogenic lines (NILs) by G1-F/G6-R primers (Supplementary Data 1). The 6373 bp genomic fragment containing the *BjuB07.CLV1* gene was then transformed into *Arabidopsis clv1* mutants (*CS45*), which produced siliques that had four locules (Figure 4a). Thirty T_1_ *Arabidopsis*-positive plants were obtained via kanamycin-resistant transformant screening, and, compared with the mutant (*CS45*) type, these T_1_ plants showed phenotypic recovery with respect to bilocular characteristics (Figures 4b,d) but different with the wild type (Figure 4c); the recovery rate was from 2.13 to 93.33%, and a recovery rate exceeding 60%was exhibited by 4 plants. A total of 27 T_2_ generations of transgenic plants from the same high-recovery T_1_ plant exhibited a 3:1 (bl:ml) segregation ratio. The average recovery rate was 63.12% (range: 28.57–92.59%). These results indicated that the *BjuB07.CLV1* gene has the same function as the *clv1* gene of *Arabidopsis* and can be stably inherited between generations. In addition, 14 T_0_ *B. juncea*-positive plants were obtained, and bilocular siliques were restored in these plants (Figures 4e–h); the recovery rate was from 17.95 to 81.58%, and a recovery rate exceeding 60% was found in 9 plants. In summary, these results indicated that the candidate gene *BjuB07.CLV1* was *BjLn2*.

### Sequence analysis

To define the gene structure, comparative sequencing between the bilocular and multilocular materials was conducted. Specific primers (G1-F/G6-R) were used to amplify the multilocular material; however, no product was observed in multilocular material. The multilocular material was subsequently amplified by segmented amplification. A 4,961 bp insertion was found in multilocular material (Supplementary Data 2, Figure 5A); this insertion resulted in a substantial increase in the full length (11,334 bp) of the *BjuB07.clv1* gene (Supplementary Data 3). The cDNAs of bilocular and multilocular materials were subsequently isolated by specific primers (Cd1F/Cd4R) and a 2,964 bp fragment was obtained from bilocular material. Comparison with the gDNA revealed that the *BjuB07.CLV1* has two exons (2,611 bp and 353 bp) and one intron (190 bp) (Supplementary Data 4). There were no products observed in the multilocular material; therefore, segmentation amplification was used. Firstly, Cd1F/Cd2R primers were used to amplify the partial fragment, and then, the rapid amplification of cDNA end (RACE) method was used to amplify the remaining cDNA of *BjuB07.clv1*. A full-length 2,349 bp cDNA of *BjuB07.clv1* was obtained (Supplementary Data 5, Figure 5B). Subsequent sequence analysis of the cDNA between *BjuB07.CLV1* and *BjuB07.clv1* revealed that a termination codon located in the *BjuB07.clv1* gene at position 2,349 bp. These results indicated that the 4,961 bp fragment insertion disrupted the normal transcription of *BjuB07.CLV1* in the multilocular material.

**Figure 5:**
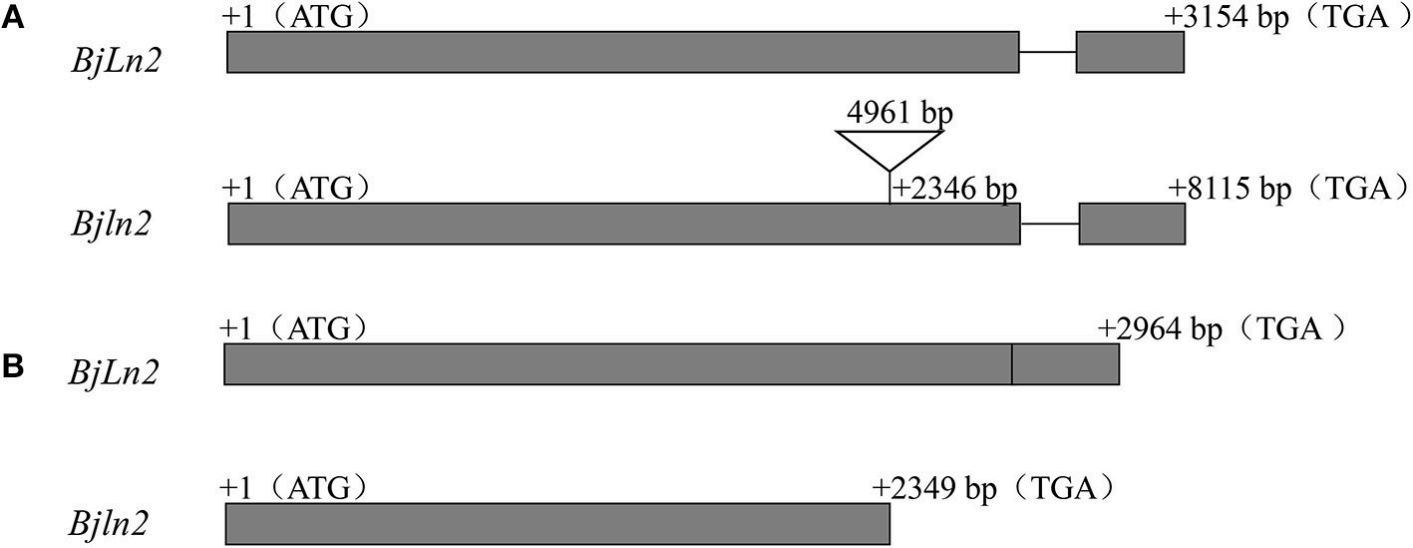
Gene structure analysis of *BjLn2* in bilocular and *Bjln2* in multilocular material. The gray boxes represent the exons, and the black lines are the introns. The initiation codon (ATG) and termination codon (TAG) are shown in the figure. The numbers in the gray box and above the black line represent the nucleotides of the exons and intron, respectively. The numbers represent the number of bases from the position to the first base of the initiation codon. **(A)** Genomic gene structure of the *BjLn2* and *Bjln2*. The black triangle represents a insertion fragment. **(B)** cDNA gene structure of *BjLn2* and *Bjln2*.

### Quantitative real-time PCR

**Figure 6:**
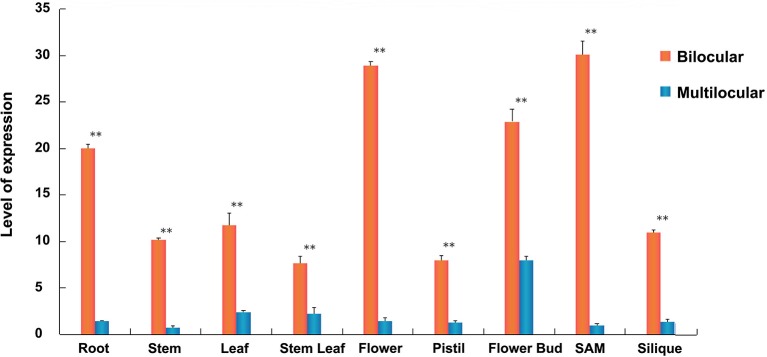
Expression analysis of *BjuB07.CLV1* gene among different tissues of bilocular and multilocular plants in BC4F5 generation by quantitative real-time PCR (qRT-PCR). The relative expression level of each tissues was normalized using primer A-Actin2 as a reference gene. The error bars report the standard deviation (3 biological replicates and 2 technical repetitions). Asterisks reprent stastictically significant differences between bilocular and multilocular, as determined by the student's *t*-test, **P < 0.01.

To explore the expression pattern of *BjuB07.CLV1* in *B. juncea*, we performed qRT-PCR tests of different tissues between homozygous bilocular and multilocular materials, such as the roots, leaves, stems, SAMs, buds, flowers, pistils, stem leaves, and siliques. The qRT-PCR results are presented in Figure 6. The *BjuB07.CLV1* gene was expressed in all tissues, and the expression levels differed significantly (*P* < 0.01) in each tissue between the bilocular and multilocular plants. The expression levels were greatest in the SAMs, flowers, and flower buds and lowest in the stem leaves and pistils. Thus, the expression pattern of the *BjuB07.CLV1* gene was not specifically localized within the plant, which means that the *BjuB07.CLV1* gene was ubiquitously expressed in all studied tissues (Figure 6).

### Histological analysis

**Figure 7:**
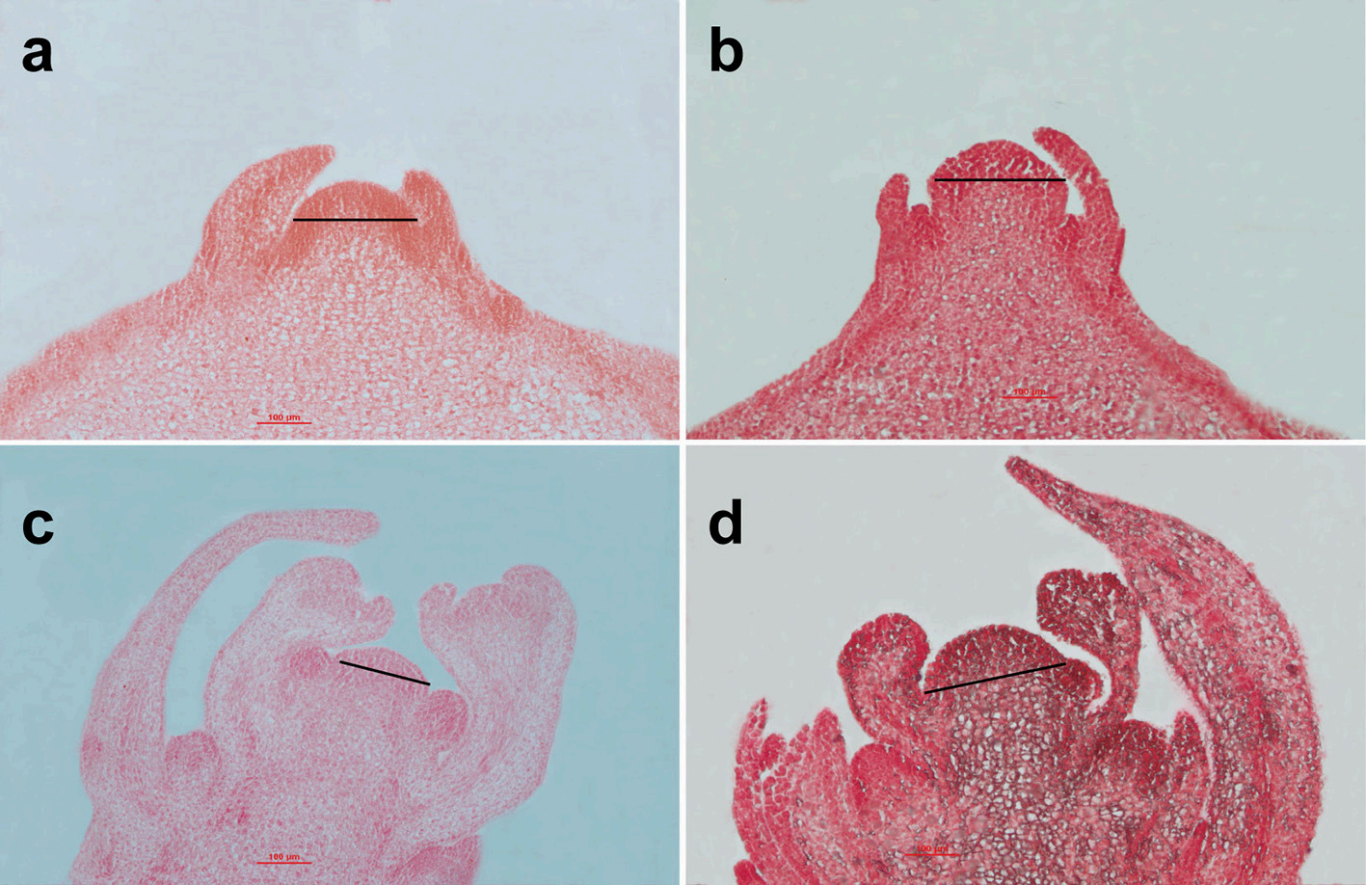
Analysis of the SAMs between homozygous bilocular and multilocular materials. **(a,c)** are the bilocular plants; **(b,d)** are the multilocular plants. **(a,b)** are SAMs of the four-leaf period; **(c,d)** are the primary inflorescence meristems.

The SAMs and flower buds of the homozygous bilocular and multilocular materials were observed. There were no significant differences in SAMs between the homozygous bilocular and multilocular materials at the four-leaf stage (Figures 7a,b), but the longitude section diameter of the primary inflorescence meristem was significantly smaller in the bilocular materials than in the multilocular materials (Figures 7c,d). These results indicated that differences of siliques occurred before the primary inflorescence meristem.

**Figure 8:**
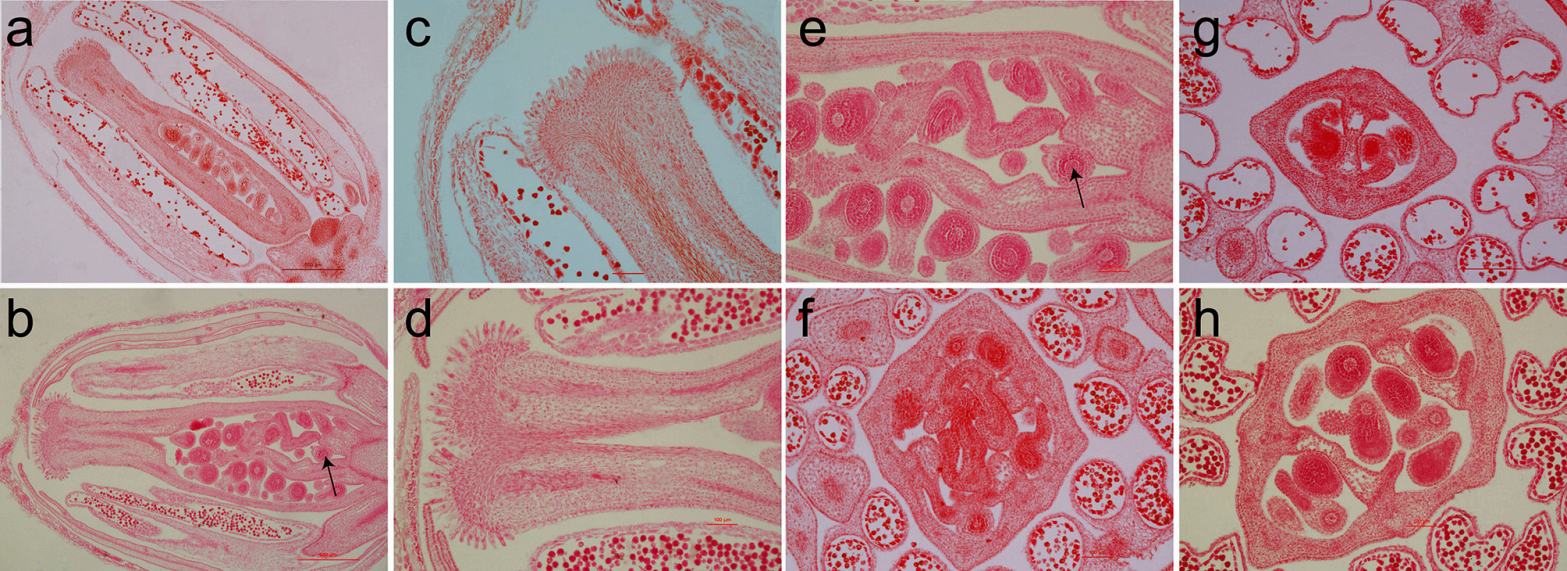
The paraffin section of the flower buds between homozygous bilocular and multilocular materials. **(a,c,g)** Are the bilocular materials. (**b,d–h)** Are the multilocular materials. **(a–e)** Are the cross section of flower buds, **(c,d)** are the stigmas. (**f–h)** are the longitudinal sections of flower buds. The arrow indicates an endogenous pistil inside the ovary.

The longitudinal section of the flower buds revealed that compared with that of the multilocular plants, the pistil of the bilocular plants had a thinner and smaller stigma (Figures 8a,b). Especially at the base, an endogenous pistil was present inside the ovary. A small number of seeds can mature in the endogenous pistil (Figures 8b,e). The stigma of the multilocular plant were also bigger than the bilocular material (Figures 8c,d). After transverse cutting the flower buds, the ovary of bilocular material was divided into two parts by a fake diaphragm, and two uniform locules were formed (Figure 8g). While the multilocular material was significantly different. Initially, the multilocular material ovary was divided into four uniform locules by two “+” shaped false septums. Meanwhile, the lengths and curvature degree of the four carpels were basically the same (Figure 8f). As the silique grows, the vast majority of the two false septums gradually show a parallel patterns, and dividing the ovary into three locules, with the middle locule was larger than the two locules on the sides, and the curvature degree of the carpels surrounding the two small locules is significantly serious than the carpels surrounding the larger locules. The cross section of the ovary was generally close to an ellipse (Figure 8h), which was similar to the view of a mature silique (Figure 4g).

## Discussion

### Phenotypic analysis

Multilocular traits have an important effect on the phenotype and yield of certain plant species, including rapeseed (Varshney, 1987; Katiyar et al., 1998; Xiao et al., 2013; Wang et al., 2016), tomato (Barrero and Tanksley, 2004; Cong et al., 2008; Muños et al., 2011) and cucumber (Zhang et al., 2015). The evolution showed that large tomatoes were due to an increase of the locule number (Muños et al., 2011). Moreover, multilocular cucumber has the potential of more grain and can be used as a elite varieties for breeding (Zhang et al., 2015). Multilocular traits may provide a new insights into rapeseed breeding. Therefore, it is necessary to study the mechanism of multilocular trait.

In this study, we found that SAMs of the multilocular plants were morphologically similar to the bilocular plants before the four-leaf period but showed significant differences at the primary inflorescence meristems, which was different with Xu et al. (2017). In the process of growth and development, the ovary of multilocular has been changing all the time. The ovary shape changed from nearly round to an oval. Meanwhile, the two false septums also went from “+” to parallel shape. We also analyzed bud paraffin sections and found that the pistil (including the stigma, style, and ovary) of the multilocular material was substantially larger than that of the bilocular material. Interestingly, there is a similar structure of pistil in the multilocular ovary. It also has a stigma, style and ovary, and can mature a small number of seeds in it. This phenotype has not been found in other multilocular crops. How do sperm get to the endogenous pistil? Whether the structure endogenous pistil is beneficial (increasing the yield) or redundant (occupying the seed-bearing space)? We are not clear. In a word, multilocular trait occurred before the primary inflorescence meristems stage.

### Cloning of the *Bjln2* by comparative mapping combined with map-based cloning

BSA, AFLP, SSR, Indel and intron polymorphism (IP) markers were used to preliminarily locate the *Bjln2* gene within the range of 3.62 cM. By expanding population individuals, the *Bjln2* gene was finally located within the interval of 0.63 cM. The sequence alignment results suggested that the *Bjln2* gene was corresponding to 11.81–16.65 Mb on B07 of *B. juncea*. Despite the collection and development of numerous markers, the *Bjln2* locus interval remained very large. It was difficult to further narrow the positioning range and predict the candidate gene via map-based cloning (Lei et al., 2007; Zhang et al., 2017). Previous studies (Lagercrantz et al., 1996; Schranz et al., 2002; Suwabe et al., 2006) have shown that genes controlling the same trait share a high degree of homology and collinearity based on mapping comparisons between *Brassica* and *Arabidopsis thaliana* and between species of *Brassica*. Genes that control these traits may be the same and may have the same evolutionary origin. Based on comparative genomic analysis, we determined that chromosomes A07 and B07 of *B. juncea* exhibit a high degree of homology within the E block (Panjabi et al., 2008; Paritosh et al., 2014). Previous studies have confirmed that the *Bjln1* locus is located within the E block of chromosome A07 (Xiao et al., 2013) and that the *Bjln2* locus is also located within the E block of chromosome B07. The *Bjln1* and *Bjln2* loci each control the same trait—that governing multilocular siliques. Based on these information, we used the sequence of *Bjln1* to develop markers, and found that the gene marker P1 was co-separated from the *Bjln2*. Markers were further developed according to the *Bjln1*. Thus, we cloned the *Bjln2* gene according to the *Bjln1* sequence by comparative mapping with map-based cloning (Xiao et al., 2018). After comparing the homology of the cloned sequence with that of *B. juncea*, we found that the *Bjln2* locus was located within the mapping interval, which further verifies the results of the comparative mapping test.

### The relationship between the *Bjua07.CLV1* gene and the *Bjub07.CLV1* gene

Previous studies have shown that multilocular siliques in *B. juncea* are controlled by two genes (*Bjln1* and *Bjln2*) (Xiao et al., 2013). The *Bjln1* gene was mapped to A07 chromosome of *B. juncea*, where the gene *Bra015812* is orthologous to *At1g75820* in *Arabidopsis* (Xiao et al., 2018). In the present study, we mapped the *Bjln2* gene to B07 chromosome of *B. juncea*, where the gene is also orthologous to *At1g75820*. These results demonstrate that the two multilocular genes (*Bjln1* and *Bjln2*) were both orthologous to the same *CLV1* gene, which encodes a putative receptor kinase that controls shoot and FM size in *Arabidopsis* (Clark et al., 1997). These results also indicated that the collinearity between chromosome A07 and chromosome B07 of *B. juncea* is very good (Panjabi et al., 2008). In addition, markers closely linked to the *Bjln2* gene were developed to provide assistance for molecular marker-assisted breeding of multilocular traits. In particular, the co-separating molecular marker P1 was obtained.

The silique phenotypes of *BjuA07.CLV1* (equivalent to *Bjln1*) and *BjuB07.CLV1* (equivalent to *Bjln2*) appeared similar, while their experssion mode was slightly different. The qRT-PCR results indicated that they were both ubiquitously expressed in all the studied tissues. The expression level of *BjuB07.CLV1* was greatest in the SAM and flower, but the expression level of *BjuA07.CLV1* was greatest in the leaf and cauline (Xiao et al., 2018).

The *BjuA07.CLV1* gene and the *BjuB07.CLV1* gene both consisted of two introns and one exon. The coding DNA sequence (CDS) of *BjuA07.CLV1* was 3,044 bp (2,611 bp intron 1, 80 bp exon and 353 bp intron 2), and the CDS of *BjuB07.CLV1* was 3,154 bp (2,611 bp intron, 1,190 bp exon and 353 bp intron 2). The sequences of the *BjuA07.CLV1* gene and the *BjuB07.CLV1* gene were more than 89% homologous. However, the homology of the *BjuA07.CLV1* is high to *B.napus*, while the homology of *BjuB.CLV1* was distant (Supplementary Figure 1). The evolution of *BjuA07.CLV1* and *BjuB07.CLV1* was different. Two amino acid (positions 28 and 63) changes as well as a 702 bp deletion in the promoter resulted in changes in the SAM and FM; *BjuA07.CLV1* was the most evolutionarily similar to *BrCLV1* (Xiao et al., 2018). However, the occurrence of multilocular traits was due to the insertion of a 4,961 bp sequence within the *BjuB07.CLV1* gene. Interestingly, the same gene or a homologous gene can produce the same phenotype despite different mutation modes. This phenomenon may be due to different evolution of the genes.

## Author contributions

DD proposed and designed this study. LX mapped and isolated the target gene. CC performed the cloning tests, complementation experiments, qRT-PCR analysis and paraffin section tests. XL participated in the gene cloning and RNA extractions.

### Conflict of interest statement

The authors declare that the research was conducted in the absence of any commercial or financial relationships that could be construed as a potential conflict of interest.
