# Supplementary material for: Comparative Mapping Combined With Map-Based Cloning of the Brassica juncea Genome Reveals a Candidate Gene for Multilocular Rapeseed
Source: Front Plant Sci. 2018 Nov 27;9:1744. doi: 10.3389/fpls.2018.01744 (PMC6277901; doi:10.3389/fpls.2018.01744)
Supplement: Supplementary file 1 [file Data_Sheet_1.PDF]

# *Supplementary Data*

**Supplementary data 1**: gDNA length of bilocular (6373 bp)

GTGTGTGGCCATTGATAGACTAGCTATGTCAAAGTAGCTTAGAACTTTCAGAAATTCTAAACTAACGACAGAT
TTTTTTTCTTACAAAGATCTAAGTGTGATATTCGAGAGTTTCCACTACGATTTTATTTATGATTAGCCACTGTTA
ACAACCAAAACTGTAAGAATATTCGAAAATTTCCCACATATTCCGAAGTTAACTCGAGCTTGTAATCAAATCA
ACGATTTTCAGAAGGTAAAAACACCATAATCTTAGCAAAAAAAAACAAAAACATAACTTTTAAAATGTCACA
AGAGAGAAGATAAGGGTAAAGTCAGACAGTTTTGCAAAACCAAAGGTAAATAACATAGAACGTGGGGACC
CTGAAGCAGAGCAGAAGAAGGTCTACAAGAAGAGTGAGTGTAGACAAAGACAAGAAAAGGAGCGTTGAC
TATTAGATGGAAGCCAAAATAGAAGGGGCAGATTTGTCTCTTTTGGAAAGGACACAGACAAACTTTCTCTAC
GGGCCATTTAATAACTAGGCCCTACTTAATAAGCCCATTAACTCCCCTCTTTGTCTTTTTAAGTTTTTAAAATAC
ATTTTCCATTTTCCTAATCACGTGACGATGAAAACAAAATGTCGTCGGATTAAACTGGTTGAGATGACAAGA
GGGCGATAAAAAGAAAGAAACCACAGCAGCTATAGCCAGGGAACGTAAAGTTAAGTCCTGTCTATCGTCTAT
AACGGTCAGTTCAGATCATAGACATTACTGTAGTAGCGTTTTCTTTCTCCCGTTACATTTTAAACGCCGTGAA
GATAGCCACTTATTTTGATATATATAGCCAAAAAGATAAACTTTCTCGATTTAAATCGTGTCAGAAGATTTAAC
AGAGGAAAGAAGACGACGACGACGTTTCCCGGTTATCCATGTCTAGTGTAGTGGCGGCTACTAACCAAATA
ACTTTTGTATATAAACTGTAAAAAAGACTATGATAAAAGTTTCTTTAAAGCACAATACAGTGGTGGATTTTTG
GGTTTGCGGTTCAATTCCAGCAAATGTTATTTGAGCACTAAACGTTAGTTAGCCGAGAAAAAAATCATAGATA
TGGTTTAGAATTCACATGATTAAATGAAATTTGACACGAAACTGATATTAAATATCGTAGTTTCAGGTTTAAAA
TTTACTAGTAACTGTAATAATTCAAAGAAAAATATTAGAAAAAATCCATGTTCGGAAACTCGGTAGGCACTAC
GCGTGCGATCGGATAACGCCTAGCACCTAATCAATTAATCAACGATTACTCGGAAATTAATCGGTGATCTTATT
TATGTTGTTTATAATATATCTCTATCATAAACCATAAAATTAGTGTGGTGCAATGGTAAGTGTTCTTTCATTTAAC
ACATGATTCCGAGTTCAAGTGCTAGGATGTGCACTTTAATAGTTTTTTATATTTTTCTTTAATGAAAGGTAAAA
CCGTTATTTATATATCATCTTCCTATTTGGGTTTTTAGATCTCTGCAATTGTAAAAACGCGATGGCTAAGAAATT
TTGACGGGTTCTAAACCAATTTTCTTTGTTTTTCTTGTTGTTTTTGTCAAAATTTCACAAATTTAAAGTAGATTA
TTAGATTTACAGGTTCAAATAAGTAAAAAATGTTGTTGTTTTTTTTTTGAATCTGATTTTCTAAGCGCATAGCT
CCAATTCGCCACGGTTGCTCACTGTATAGCGATTTCTCAGCCGCTAAATCAGCTAGGCGGTCGAGTTTTAGAA
CATGGGAAAAAATAGAATGCGAGATATAAAAAGACAATGAACGATTGACACATGAATGTTGACTGCTAGTCG
TAGTTCATATCTAGAAGCTGAATGTCCTTTTAACCATTTATAACTTTATATCCGTATGATAATTTCATAAATCTACC
TCGATAGAGAGTAACCATCACAATTTTTTTTCCAGTAATTACGAGATTCATTGGAGAAAAAAAAAGGGTTTTT
AAAGCGCAATATTTCTAGTGGTATATTTATTCTCATTCAAGGTCATATAAAAAAAGATTAAAAAAGCAATAATAT
TCTCTCTGAAAGTCTAGAGGTATTTTAGAACAGTACTGCTACGAATCTCTTCTTCATCTTCATAATATGCCATTG
TGATTGTTCATATATATCTGTGCACTCATCCTATCGCATAAAACTCACGCTAACTTCTTATTCTCTCTCTCTTTCTC
TGAGAAGACAGCTTTGAAGAAAGAAAGAAAAAAATGGAGATGAGACTTTTGAAAACACACCTTCTGTTTCT
GCATCTTCACTACGTTATCTCGATTTTGCTTCTATGTTTCTCACCATGCTTCGCTTCCACTGACATGGACCATCT
CCTCAACCTCAAATCCTCAATGACTGGTCACAACGGACACGGTCTCCACGACTGGGTTCACTCCACTTCTCCC
ACGGCTCACTGTTCTTTCTCCGGCGTCTCATGCGACGCCGACGCTCGTGTTGTCTCACTCAACGTCTCTTTCA
CTCCTCTGTTCGGAACCATCTCACCGGAGATTGGGATGCTGAACCGTTTGGTGAATCTGACGTTAGCCGCGA
ATAACTTCTCCGGTAGGTTGCCGCTGGAGATGAAGAGTCTCACTTCACTAAAGGTTCTCAACATCTCCAACA
ACGTAAACCTCAACGGAACCTTCCCCGGAGAGATTCTCACTTCCATGGTCGACCTCGAAGTCCTCGACGCCT
ATAACAACAACTTCACTGGGCCGTTACCGCCGGAGATTCCCGGGCTGAACAAGCTCAAACACCTCTCTCTCG
GAGGAAACTTCTTCACCGGAGAGATTCCCGAGAGTTACGGAGATATCCAAAGCTTGGAGTATCTCGGCCTC

AACGGAGCCGGACTCTCCGGCGAATCTCCGGCGTTCTTGTCACGTCTCAAGAATCTCAGAGAAATGTACGTC
GGCTACTTCAACAGCTACACCGGCGGCGTTCCGCCGGAGTTCGGCGAATTAACGAAGCTAGAAATCCTCGA
CATGGCGAGCTGTACTCTCACCGGAGAGATTCCGACGACACTGAGTAATCTGAAACACTTGCACACGCTGTT
CCTCCACATCAACAACTTAACCGGAAACATCCCACCAGAACTCTCCGGTTTAATCAGCTTAAAATCTCTCGAT
CTATCAATTAACCAGCTAACCGGAGAGATTCCTCAGAGCTTCATCTCTCTGGGAAACATCACTCTCATCAACC
TCTTCAGAAACAACCTCCACGGGCCGATACCGGAGTTCATCGGAGAAATGCCGAACCTCCAAGTCTTCCAG
GTGTGGGAGAACAACTTCACGTTAGAGTTACCGGCGAATATCGGCCGGAACGGGAATCTGAAAAAGCTCG
ACGTCTCTGAAAACCATCTCACCGGACTCATCCCCGTGGATTTATGCAGAGGTGGGAAGCTGGAGGTTCTG
GTACTCTCCAACAACTTCTTCTTCGGCTCCATCCCGGAGAAGCTAGGTCAATGCAAATCTCTAAACAAGATCA
GAATCGTCAAGAATCTCCTCAACGGGACCGTTCCGGCGGGACTATTCAACTTACCACTCGTTACAATCATCGA
GCTCGCGGATAACTTCTTCTCCGGGGAACTTCCTACGGAGATGTCCGGCGACGTTCTCGATCATATCTACCTA
TCTAACAACTGGTTTACCGGTTTAATCCCTCCGGCTATCGGTAATTTCAAAAATCTACAGGATCTGTTCTTAGA
CCGGAACCGGTTTAGCGGGAATATTCCGAGAGAAGTTTTCGAGCTGAAGCATCTCACGAAGATCAACACGA
GCGCTAACAACCTAACCGGCGATATCCCTGACTCATTCTCGCGATGCACTTCCTTAATCTCCGTCGATCTCAGC
CGTAACAGAATCGGCGGAGATATCCCGAAAGACATCCACGACGTGATTAACTTAGGAACTCTCAATCTCTCC
GGGAATCAGCTCACCGGCTCGATCCCGATCGGAATCGGGAAGATGACGAGCTTAACCACTCTCGATCTCTCC
TTCAACGACCTCTCCGGGAGAGTACCACTCGGCGGTCAGTTCCTAGTCTTCAACGACACTTCCTTCGCCGGA
AACCCTTACCTCTGCCTCCCTCACCACGCCTCCTGCCTAACGCGTCCAGGACAAACCTCTGATCGCATCCACG
CGGCGCTGTTCTCTCCGTCGAGGATCGTCATCACGATCATCGCGGCGATCACGGCGTTGATCCTCATCAGCGT
CGCGATCCGTCAGATGAACAAGAAGAAACACGAGAGATCCCTCTCGTGGAAGCTAACCGCCTTCCAGCGAC
TCGATTTCAAGGCGGAAGACGTCCTCGAGTGCCTTCAAGAAGAGAACATAATCGGCAAAGGCGGAGCGGG
GATCGTCTACCGCGGATCCATGCCGAACAACGTAGACGTCGCGATCAAACGGTTAGTTGGACGCGGAACGG
GGAGGAGCGATCACGGATTCACGGCGGAGATTCAGACGTTAGGAAGAATCCGCCACCGTCATATAGTGAGA
CTACTCGGATACGTGGCGAACAAGGACACGAACCTGCTTCTATACGAGTACATGCCTAACGGGAGCCTCGGC
GAGCTTTTGCACGGGTCTAAAGGCGGTCATCTTCAGTGGGAGACGAGGCACAGAGTAGCCGTTGAAGCGG
CGAAAGGACTGTGTTATCTTCACCATGACTGTTCGCCGTTGATCTTGCACAGAGACGTTAAGTCCAATAACAT
TCTCCTGGACTCTGATTTCGAAGCCCATGTTGCTGATTTTGGGCTTGCTAAGTTCTTAGTGGACGGTGCTGCT
TCTGAGTGTATGTCCTCAATAGCTGGCTCCTACGGTTACATCGCTCCAGGTTAGTTTAAACGTGTTTTATATAA
CAAACTATGCCATGTTCTTTAAATGTTACTGAGACTAATCATATGTTAGAGTTTGTTGTCCGTTTTATTGTCGTA
TAGCTGATTACTATGATTTGTGACACACAAATGAATGTGACTTTAACAACTAAAATATGTACTAATCATTGTTTG
TTTTGGGTTTGATAGAGTATGCATACACTCTCAAAGTAGACGAGAAGAGTGATGTGTATAGCTTTGGAGTGG
TCTTGTTGGAGCTGATAGCTGGGAAGAAACCGGTTGGTGAGTTTGGGGAAGGAGTGGATATAGTGAGGTG
GGTGAGGAACACGGAGGGTGAGATACCTCAGCCTTCGGATGCAGCTACTGTTGTTGCGATCGTTGACCCGA
GGTTGACTGGTTACCCGTTGACCAGTGTGATTCATGTGTTCAAGATAGCGATGATGTGTGTGGAGGATGAGG
CCGCGTCAAGGCCTACGATGAGGGAAGTTGTGCACATGCTAACTAACTCTCCTAAGTCCGTGACTAACTTGA
TCGCCTTCTGAGTTCTGACCCAATCAAAGGCTAATGAAAAAATAAGATTGTGTTGTGTGTAATGATAATATTTT
TGTTTTTTTAAGTGGACATATATCGTTTTAAATTATTGCCGAGTTGTATGTATATTTGCTGTGTGTTGGATCTGTA
TATCGCCTCTTATGTGTTTGTTATCTACTCTTAAGTCTGTAATGAGATTCTTATGCTTATTGATGATGGCTTTTAA
ATTTTAGCTATTTCTTCTTTCAGACAAAGTTGCGTAAGTAATGAAAAGAGATAAAAACCAAGAAGCATATGTT
CATTACAAGCAGACTCATCATTTGTATTATCAACAAGAGCAGACAAACATGTGTCTTAGATCAAAGATGATGA
TGATGATCTGGAGCCAATCCAAATGAGATCCACTCCACTTTTTCCTCTCACAAACAATTTCATCTGCTAGATTC
TACCAATATCCAACTCGAAATCTAAATATAGATTCACATGTTCATTATGGTAATAGACCCAAGATCCTCCATTAC
AATGTGGGATGTTCTCTCTACACCAACAAGCCCATACAAATTACACTCAAATCCTTTAACACAGCTCTTAAATT
CAAAATCATTTTTTATCAAAAAAAAAAAAAACAAGAACATTATTCATCATCGCTATAACACTAAAAAAAGAAAC

ACAGACATAATTTTCATAGCTACAATAAATAACACCAATGACTAATTCCTCAATAGCTGTTATATACTTAGAAAG
CACCGTAAGGTATGTGTTGAGGAAGCGTGAATCGAACCTGCGAAGCTGGTAACCGAACCGCAACGCCGTG
CCAGCAAATCCCTCCTTTACAAATTTCACCACCTTGGTGATGCTGAAGGTGACTAAGATTCTCAACGCCTACG
CGATTGTACCGCGAAACCGCATCCGCCGCCCTGCGAGTCACGTCCCAAGCCAGCGTGACCGCAAGCAAAAC
TCCGACCGTCGTAATCGAAACTACAACGACGAATCCA

**Supplementary data 2**: Insertion sequence in multilocular (4961bp)

TGAGAACAAGAGGAGACAGAGAGAATTTTAATAAAACTTCCTTTTGTATTCACTTTCTCAAAACGTTTCTTTA
CAAGATAATCGAGTATATATACAACCTCAAGATAAACCCTAGTTCCCTTAACTTTGGACCACTCAACACGTGTC
TTCGTCACACACACTCTACACCTTGCAAGGCATCGTAACGGCTAGACAAAGCTATTCCACAGTTTACTCCAGC
TGGTGCATTTGTCTTCTTGTTCTGTACTTGTACGAACTCTTCATTACTCTGTTTCAGAGTTTGTCTTTGGGCCA
GCATACTTCCTTCAGTTAATGGACCAGTCGCTGATATACCAAGGCCCATCATGGCTTGTTTACTGTCATCCCCC
CGCAAACTGATGATGGGTGGAGCACCAACACTCAGTTTGGCTCTGAGACTCTGAAAGGAATGGCGTGGGA
GAGATTTAGTGAAGATATCTGCAAGTTGCAGTGCAGAAGGAATGTGCTGAACCTCCAAGACACCAAGAGCC
ACCCGCTCCCTAACATAATGGTAATCCGTTTCAATATGTTTGGACCGACCGTGAAGAACCGGGTTTGCTGTAA
GATAAACTGCAGAGAGGTTATCACAGTGAAGAACAGCCGGCTTTGGTTGTTTGATCTTCATATCACGCATCA
CAAACGAAATCCATGTTAACTCAGTCCGTCTTGCAGAGCTCGATATTCAGCCTCAGTGGACGACCGCGAGAC
AGTCGGTTGGCGCTTTGCCGACCAGGAGACAATGTTAGAACCAATCATGACACAAAAACCACCAGTCGACC
GTCTTGTCTCCCTACACCCTGCCCAGTCACTATCACTATACGCGACAACATCCAAACCAGAGTTCTTACGGAA
GTAAATCCCCATGGATGAAGTACCACGAAGGTAGCGAAGAACCCGCTTCAACAAATGAAAGTCAGCTTCAG
TGGGAGAATGCATTCTCTGACAAATGAAATTCACAGCAAACTGAATGTCGGGTCGAGTGATAGTGAGATACT
GTAACTTCCCAGCAAGACTCCGAAAGTAGCTAGGCTCAGGGAACGGCCGAGTATCTTCATAAACTTGATCGA
GTCGCAAAGGGAGAGGTGTATGAACAGGATTACACGCAGACATATTGGCATGGTGAAGAATCTCTTCAGTG
TACTTGGATTGAGAGAGGAAAAGACCAGAATCTGTAATCTGAGCCTGAACGCCCAAGAAGTAGTGCAGATT
ACCCAAATCTTTCATGGAGAATCGGCTACTGAGCTCAGTGATAAGGGACTTGAACAAGGATGGATTTGAGC
CAGTGAGAAGTATATCATCCACATAAAGGAGGAGAACTAATGTGTCGCCATTGTGATGATAAACAAAGAGAG
AGGGATCAGCTTTACTGCAGACAAAGCCATACTCAAGAAGGAAGTTACTAAATTTATCAAACCAAGCCCGTG
GAGCTTGTTTTAAACCGTACAGAGCCTTCTTGAGAGAGCACACATGATGAGGTTTAGTGCAATCTTCAAAGC
CGGGTGGTTGTATCATATAGACTGCTTCTTGAAGATCTCCATGAAGGAAAGCATTTTTAACATCAAGCTGAGT
GATGTTCCAGTTCTTTGAAGCAGCTACAGCAAGAACTATCCGAATGGTAGAAGTCCTAACCACTGGACTGTA
TGTCTCTGTGAAATCTACTCCTTCTTCTTGATCAAACCCCTTAGCAACTAACCGCGCTTTAAGTTTATTGACTG
TCCCATCTGCGTTAAGCTTCACTGTAAAAACCCATCTGCAACCAAGAACATTCATAGTTTCTGTAGCTTCCGTT
AAAACCCAAGTGTGGTTCTGATAAATCGAGTCTAGTTCATCCAACATTGCTTGACGCCATCCAGGGTGCGCC
AAAGCTTCAGATATAGTCTGTGGTAAGGAGGGAATCGTCTTGCTTGTGACCAGAGCGTAACGTGGATTAGG
TTTGCTAATACCAGCTCGTGATCTCGTCATCATAGGATGACTAGGCGCTGCTACGTCTGGTGAGGCACGAGG
AGGTGATTGAGCAACCTCAGGAAGACTTGTGGTGGATACGTCGCCTTCAGACAGCTCGAAGTCTTCCTGCC
CATGATCAGAACGAGATGGGGTTGCAATAGGCGTATCTTGACCCGTGGTGATAGGAGTATCCTGTTGAGGAT
ACTCTGTAACTTGAGGACACTCTGTAACTTGAGAAGGTACTGTAGGCAGATAGACCGGAGGAGCTTCAAAT
GCAGTACCATCTTGCCAAGCTTTAAGCAAACCAGAGTTGTGTTTCACAAGCAGATGTTTATACTGTCCAGTG
AATGGGAAGGTCTGCTCATCGAAGATAACGTGGCGAGAGATGTAAACTCGACCTGTTGGTGGATGCAAACA
CCTGTAACCTTTGTACTGAGGATGATAACCGAGGAAGACACACTGTAAGGATCGGGGCTCGAACTTGTGTT
GAGCATAGGGACGCAAGCACGGATAGCACGCAGAGCCAAAGATTCTGAGAAATGAGTAGTCTGGTGCTTT
ACTAGAGAGAACCTCTGAAGGGCTTTTGTTGTTCAGAACAGAAGAAGGGATCATGTTGCTGATGAAGTTAG
CAGAGTAGAAGGCCTCAACCCAATATTTAAGAGGAGTGTGACTTTGAAATAACATAGAAAGACCCAATTCAG

TAAGATGTCTATGTTTTCTCTCAGCAATACCATTTTGTTGAGGAGTTGAAGGACATGAAACAAGATGAACAAT
CCCTTGATCTCTGAGATGATTGCGAAACCTTGTGCTCATAAACTCACCACCCCCATCGCTCTGAAAGACTTTC
AGTTTCTTGTTAAACTGATTTTCGATTTGTTTTTGAAAGACAATGAACACATCACAAAAATCAGACTTGGCTT
TAAGAGGTATTATCCATGAGTAGCGACTAAAGTTGTCAACAAAGACTGCATAATACTTGAACCCTTGAACTGA
TACAACCGGAGAAGGACCCCACAGATCACAATGTATCCGATCTATGGGTTCCTTGACACTAGACTCTGAAATA
AAAAAAGGAAGCTGACTGCTCTTTCCCATCTGGCAAGGTCCACAAACGGGAGAAATGCTGCTCTTATTGACT
ATGATTGCCTTGCTTTGCTTGAGGAGCTGAAGAATCTGGAAGTTCGCGTGCCCAAGTCGCTGATGCCACACC
ATATCACTAGCTGCACACTGACGATTGGAGAAGTAAGCCGCAAACTCCTTCTTCTCCAGCACATACAAGCCTC
TATTTCGAGTTCCCTTTGTCACCACCTTCTGAGCATCGAGGTCGATGACATAGACAGCATTAGAGTCAAAGAA
CACCCCACAGGGGTAATCATCACACAGTTTGGATACAGATAATAGTGATTTTTGCATTGAAGGACAGACAAG
AACATCGTTTAGAGGAAGGCTACCTGTCTCAGTGGTGAGCGTTGTGGAACCAACGTGGGTAATCGGAAGG
AAATTGCCATCTGCCACCATAACATGCTCAGAACCATTGTATGGAGTAGTGTTCTGCATCTGAGTTGTATTAGG
AACCATGTGAGCAGTGGCACCTGAGTCAGCTATCCATTCCCTGCCACTAGGATCAGAGACTGGAAAAGCGG
ACAGAGCATGAGGAACATCGTCACTCTGATAAGAGTTATCAAAGCGGTTCCAGCAACGAAGAGCGGAGTG
GCCGGTGCGGCCACAGATCTGACACACAGGGCGAGAGTTGTTGTTGTTATTGCCACCACCAGAGTTGCTGA
CTTGTTGAGTGAAACCTCTTCCACGAGTTGAGAAGCCACCACGACCAGAATAGCCACCGCGACCACGAGAA
TAATTATTGCTTCGTCCTCTGTTCGAGTTGTTGTAGTACCCTCGGTGAGTCTGGAAAGCCATGTTGGGAGTAA
CATCTGATGTTGCTTCATACGACTGAAGACGAGCGTCAAAACCAGAGACATCAGAAATCACATCGTTCAAAG
TCGGAGGAGGGATTCGAGTCATGGAGCTCTGAACGACAGTGATAATTGGATCATATTCTCTTCCAAGGCCAT
TGATAAATGAGAAGATCTTCATGGATTCCTCTATAGGCTTCCCAATGGAGCTCAACTTGTCACAAATGGCTCG
AAACTCACGACAGTAGGTAGTAAAGTCTTTACCTTTCTTAGTCATCAACTGGAGAGACCGTCTGAGCTCAAA
CTCACGAGCAATAGAACTCTTGTTGTAGTTGTCAGCTAACGATAACCAGACATCACGAGATGTAGATAGACCA
TGTACTGTACCAAGGACTTCCTCAGTAAGTGTTCCAAAGATCCAAGAAATGACGAGTTGATCCGTACAAGTC
CAAGCTTCAAAGCGAGGATTTGGGGCTTGAACGTTGACGTTGTCGACCACGGTTGTGACTAGCTCCGGCG
GAGGTGGAATCTGACCAGTAGCAAAGCCCAACAGCTTCTGACTGCGAAGAAGAGACTCCATTTGAGTCTTC
CATAGGAGATAGTTGCTGTCACTCAGTTTAATGGTAACAGAACTTGTAATGTGAACACTGGTGGGAAAAGG
GTATGGATCTTGCTCAGCCATGACAGCACCTGTTAGGGTTAAATGAGCTCTGATACCATGAGAACAAGAGGA
GACAGAGAGAATTTTAATAAAACTTCCTTTTGTATTCACTTTCTCAAAACGTTTCTTTACAAGATAATCGAGTA
TATATACAACCTCAAGATAAACCCTAGTTCCCTTAACTTTGGACCACTCAACACGTGTCTTCGTCACACACACT
CTACACCTTGCAAGGCATCGTAACGGCTAGACAAAGCTATTCCACAGTTTACTCCAGCTGGTGCATTTGTCTT
CTTGTTCTGTACTTGTACGAACTCTTCATTACTCTGTTTCAGAGTTTGTCTTTGGGCCAGCATACTTCCTTCAG
TTAATGGACCAGTCGCTGATATACCAAGGCCCATCATGGCTTGTTTACTGTCATCGGC

**Supplementary data 3**: gDNA length of multilocular (11334 bp)

GTGTGTGGCCATTGATAGACTAGCTATGTCAAAGTAGCTTAGAACTTTCAGAAATTCTAAACTAACGACAGAT
TTTTTTTCTTACAAAGATCTAAGTGTGATATTCGAGAGTTTCCACTACGATTTTATTTATGATTAGCCACTGTTA
ACAACCAAAACTGTAAGAATATTCGAAAATTTCCCACATATTCCGAAGTTAACTCGAGCTTGTAATCAAATCA
ACGATTTTCAGAAGGTAAAAACACCATAATCTTAGCAAAAAAAAACAAAAACATAACTTTTAAAATGTCACA
AGAGAGAAGATAAGGGTAAAGTCAGACAGTTTTGCAAAACCAAAGGTAAATAACATAGAACGTGGGGACC
CTGAAGCAGAGCAGAAGAAGGTCTACAAGAAGAGTGAGTGTAGACAAAGACAAGAAAAGGAGCGTTGAC
TATTAGATGGAAGCCAAAATAGAAGGGGCAGATTTGTCTCTTTTGGAAAGGACACAGACAAACTTTCTCTAC
GGGCCATTTAATAACTAGGCCCTACTTAATAAGCCCATTAACTCCCCTCTTTGTCTTTTTAAGTTTTTAAAATAC
ATTTTCCATTTTCCTAATCACGTGACGATGAAAACAAAATGTCGTCGGATTAAACTGGTTGAGATGACAAGA
GGGCGATAAAAAGAAAGAAACCACAGCAGCTATAGCCAGGGAACGTAAAGTTAAGTCCTGTCTATCGTCTAT

AACGGTCAGTTCAGATCATAGACATTACTGTAGTAGCGTTTTCTTTCTCCCGTTACATTTTAAACGCCGTGAA
GATAGCCACTTATTTTGATATATATAGCCAAAAAGATAAACTTTCTCGATTTAAATCGTGTCAGAAGATTTAAC
AGAGGAAAGAAGACGACGACGACGTTTCCCGGTTATCCATGTCTAGTGTAGTGGCGGCTACTAACCAAATA
ACTTTTGTATATAAACTGTAAAAAAGACTATGATAAAAGTTTCTTTAAAGCACAATACAGTGGTGGATTTTTG
GGTTTGCGGTTCAATTCCAGCAAATGTTATTTGAGCACTAAACGTTAGTTAGCCGAGAAAAAAATCATAGATA
TGGTTTAGAATTCACATGATTAAATGAAATTTGACACGAAACTGATATTAAATATCGTAGTTTCAGGTTTAAAA
TTTACTAGTAACTGTAATAATTCAAAGAAAAATATTAGAAAAAATCCATGTTCGGAAACTCGGTAGGCACTAC
GCGTGCGATCGGATAACGCCTAGCACCTAATCAATTAATCAACGATTACTCGGAAATTAATCGGTGATCTTATT
TATGTTGTTTATAATATATCTCTATCATAAACCATAAAATTAGTGTGGTGCAATGGTAAGTGTTCTTTCATTTAAC
ACATGATTCCGAGTTCAAGTGCTAGGATGTGCACTTTAATAGTTTTTTATATTTTTCTTTAATGAAAGGTAAAA
CCGTTATTTATATATCATCTTCCTATTTGGGTTTTTAGATCTCTGCAATTGTAAAAACGCGATGGCTAAGAAATT
TTGACGGGTTCTAAACCAATTTTCTTTGTTTTTCTTGTTGTTTTTGTCAAAATTTCACAAATTTAAAGTAGATTA
TTAGATTTACAGGTTCAAATAAGTAAAAAATGTTGTTGTTTTTTTTTTGAATCTGATTTTCTAAGCGCATAGCT
CCAATTCGCCACGGTTGCTCACTGTATAGCGATTTCTCAGCCGCTAAATCAGCTAGGCGGTCGAGTTTTAGAA
CATGGGAAAAAATAGAATGCGAGATATAAAAAGACAATGAACGATTGACACATGAATGTTGACTGCTAGTCG
TAGTTCATATCTAGAAGCTGAATGTCCTTTTAACCATTTATAACTTTATATCCGTATGATAATTTCATAAATCTACC
TCGATAGAGAGTAACCATCACAATTTTTTTTCCAGTAATTACGAGATTCATTGGAGAAAAAAAAAGGGTTTTT
AAAGCGCAATATTTCTAGTGGTATATTTATTCTCATTCAAGGTCATATAAAAAAAGATTAAAAAAGCAATAATAT
TCTCTCTGAAAGTCTAGAGGTATTTTAGAACAGTACTGCTACGAATCTCTTCTTCATCTTCATAATATGCCATTG
TGATTGTTCATATATATCTGTGCACTCATCCTATCGCATAAAACTCACGCTAACTTCTTATTCTCTCTCTCTTTCTC
TGAGAAGACAGCTTTGAAGAAAGAAAGAAAAAATGGAGATGAGACTTTTGAAAACACACCTTCTGTTTCT
GCATCTTCACTACGTTATCTCGATTTTGCTTCTATGTTTCTCACCATGCTTCGCTTCCACTGACATGGACCATCT
CCTCAACCTCAAATCCTCAATGACTGGTCACAACGGACACGGTCTCCACGACTGGGTTCACTCCACTTCTCCC
ACGGCTCACTGTTCTTTCTCCGGCGTCTCATGCGACGCCGACGCTCGTGTTGTCTCACTCAACGTCTCTTTCA
CTCCTCTGTTCGGAACCATCTCACCGGAGATTGGGATGCTGAACCGTTTGGTGAATCTGACGTTAGCCGCGA
ATAACTTCTCCGGTAGGTTGCCGCTGGAGATGAAGAGTCTCACTTCACTAAAGGTTCTCAACATCTCCAACA
ACGTAAACCTCAACGGAACCTTCCCCGGAGAGATTCTCACTTCCATGGTCGACCTCGAAGTCCTCGACGCCT
ATAACAACAACTTCACTGGGCCGTTACCGCCGGAGATTCCCGGGCTGAACAAGCTCAAACACCTCTCTCTCG
GAGGAAACTTCTTCACCGGAGAGATTCCCGAGAGTTACGGAGATATCCAAAGCTTGGAGTATCTCGGCCTC
AACGGAGCCGGACTCTCCGGCGAATCTCCGGCGTTCTTGTCACGTCTCAAGAATCTCAGAGAAATGTACGTC
GGCTACTTCAACAGCTACACCGGCGGCGTTCCGCCGGAGTTCGGCGAATTAACGAAGCTAGAAATCCTCGA
CATGGCGAGCTGTACTCTCACCGGAGAGATTCCGACGACACTGAGTAATCTGAAACACTTGCACACGCTGTT
CCTCCACATCAACAACTTAACCGGAAACATCCCACCAGAACTCTCCGGTTTAATCAGCTTAAAATCTCTCGAT
CTATCAATTAACCAGCTAACCGGAGAGATTCCTCAGAGCTTCATCTCTCTGGGAAACATCACTCTCATCAACC
TCTTCAGAAACAACCTCCACGGGCCGATACCGGAGTTCATCGGAGAAATGCCGAACCTCCAAGTCTTCCAG
GTGTGGGAGAACAACTTCACGTTAGAGTTACCGGCGAATATCGGCCGGAACGGGAATCTGAAAAAGCTCG
ACGTCTCTGAAAACCATCTCACCGGACTCATCCCCGTGGATTTATGCAGAGGTGGGAAGCTGGAGGTTCTG
GTACTCTCCAACAACTTCTTCTTCGGCTCCATCCCGGAGAAGCTAGGTCAATGCAAATCTCTAAACAAGATCA
GAATCGTCAAGAATCTCCTCAACGGGACCGTTCCGGCGGGACTATTCAACTTACCACTCGTTACAATCATCGA
GCTCGCGGATAACTTCTTCTCCGGGGAACTTCCTACGGAGATGTCCGGCGACGTTCTCGATCATATCTACCTA
TCTAACAACTGGTTTACCGGTTTAATCCCTCCGGCTATCGGTAATTTCAAAAATCTACAGGATCTGTTCTTAGA
CCGGAACCGGTTTAGCGGGAATATTCCGAGAGAAGTTTTCGAGCTGAAGCATCTCACGAAGATCAACACGA
GCGCTAACAACCTAACCGGCGATATCCCTGACTCATTCTCGCGATGCACTTCCTTAATCTCCGTCGATCTCAGC
CGTAACAGAATCGGCGGAGATATCCCGAAAGACATCCACGACGTGATTAACTTAGGAACTCTCAATCTCTCC

GGGAATCAGCTCACCGGCTCGATCCCGATCGGAATCGGGAAGATGACGAGCTTAACCACTCTCGATCTCTCC
TTCAACGACCTCTCCGGGAGAGTACCACTCGGCGGTCAGTTCCTAGTCTTCAACGACACTTCCTTCGCCGGA
AACCCTTACCTCTGCCTCCCTCACCACGCCTCCTGCCTAACGCGTCCAGGACAAACCTCTGATCGCATCCACG
CGGCGCTGTTCTCTCCGTCGAGGATCGTCATCACGATCATCGCGGCGATCACGGCGTTGATCCTCATCAGCGT
CGCGATCCGTCAGATGAACAAGAAGAAACACGAGAGATCCCTCTCGTGGAAGCTAACCGCCTTCCAGCGAC
TCGATTTCAAGGCGGAAGACGTCCTCGAGTGCCTTCAAGAAGAGAACATAATCGGCAAAGGCGGAGCGGG
GATCGTCTACCGCGGATCCATGCCGAACAACGTAGACGTCGCGATCAAACGGTTAGTTGGACGCGGAACGG
GGAGGAGCGATCACGGATTCACGGCGGAGATTCAGACGTTAGGAAGAATCCGCCACCGTCATATAGTGAGA
CTACTCGGATACGTGGCGAACAAGGACACGAACCTGCTTCTATACGAGTACATGCCTAACGGGAGCCTCGG
CTGAGAACAAGAGGAGACAGAGAGAATTTTAATAAAACTTCCTTTTGTATTCACTTTCTCAAAACGTTTCTTT
ACAAGATAATCGAGTATATATACAACCTCAAGATAAACCCTAGTTCCCTTAACTTTGGACCACTCAACACGTGT
CTTCGTCACACACACTCTACACCTTGCAAGGCATCGTAACGGCTAGACAAAGCTATTCCACAGTTTACTCCAG
CTGGTGCATTTGTCTTCTTGTTCTGTACTTGTACGAACTCTTCATTACTCTGTTTCAGAGTTTGTCTTTGGGCC
AGCATACTTCCTTCAGTTAATGGACCAGTCGCTGATATACCAAGGCCCATCATGGCTTGTTTACTGTCATCCCC
CCGCAAACTGATGATGGGTGGAGCACCAACACTCAGTTTGGCTCTGAGACTCTGAAAGGAATGGCGTGGG
AGAGATTTAGTGAAGATATCTGCAAGTTGCAGTGCAGAAGGAATGTGCTGAACCTCCAAGACACCAAGAGC
CACCCGCTCCCTAACATAATGGTAATCCGTTTCAATATGTTTGGACCGACCGTGAAGAACCGGGTTTGCTGTA
AGATAAACTGCAGAGAGGTTATCACAGTGAAGAACAGCCGGCTTTGGTTGTTTGATCTTCATATCACGCATC
ACAAACGAAATCCATGTTAACTCAGTCCGTCTTGCAGAGCTCGATATTCAGCCTCAGTGGACGACCGCGAGA
CAGTCGGTTGGCGCTTTGCCGACCAGGAGACAATGTTAGAACCAATCATGACACAAAAACCACCAGTCGAC
CGTCTTGTCTCCCTACACCCTGCCCAGTCACTATCACTATACGCGACAACATCCAAACCAGAGTTCTTACGGA
AGTAAATCCCCATGGATGAAGTACCACGAAGGTAGCGAAGAACCCGCTTCAACAAATGAAAGTCAGCTTCA
GTGGGAGAATGCATTCTCTGACAAATGAAATTCACAGCAAACTGAATGTCGGGTCGAGTGATAGTGAGATA
CTGTAACTTCCCAGCAAGACTCCGAAAGTAGCTAGGCTCAGGGAACGGCCGAGTATCTTCATAAACTTGATC
GAGTCGCAAAGGGAGAGGTGTATGAACAGGATTACACGCAGACATATTGGCATGGTGAAGAATCTCTTCAG
TGTACTTGGATTGAGAGAGGAAAAGACCAGAATCTGTAATCTGAGCCTGAACGCCCAAGAAGTAGTGCAGA
TTACCCAAATCTTTCATGGAGAATCGGCTACTGAGCTCAGTGATAAGGGACTTGAACAAGGATGGATTTGAG
CCAGTGAGAAGTATATCATCCACATAAAGGAGGAGAACTAATGTGTCGCCATTGTGATGATAAACAAAGAGA
GAGGGATCAGCTTTACTGCAGACAAAGCCATACTCAAGAAGGAAGTTACTAAATTTATCAAACCAAGCCCGT
GGAGCTTGTTTTAAACCGTACAGAGCCTTCTTGAGAGAGCACACATGATGAGGTTTAGTGCAATCTTCAAAG
CCGGGTGGTTGTATCATATAGACTGCTTCTTGAAGATCTCCATGAAGGAAAGCATTTTTAACATCAAGCTGAG
TGATGTTCCAGTTCTTTGAAGCAGCTACAGCAAGAACTATCCGAATGGTAGAAGTCCTAACCACTGGACTGT
ATGTCTCTGTGAAATCTACTCCTTCTTCTTGATCAAACCCCTTAGCAACTAACCGCGCTTTAAGTTTATTGACT
GTCCCATCTGCGTTAAGCTTCACTGTAAAAACCCATCTGCAACCAAGAACATTCATAGTTTCTGTAGCTTCCG
TTAAAACCCAAGTGTGGTTCTGATAAATCGAGTCTAGTTCATCCAACATTGCTTGACGCCATCCAGGGTGCGC
CAAAGCTTCAGATATAGTCTGTGGTAAGGAGGGAATCGTCTTGCTTGTGACCAGAGCGTAACGTGGATTAG
GTTTGCTAATACCAGCTCGTGATCTCGTCATCATAGGATGACTAGGCGCTGCTACGTCTGGTGAGGCACGAG
GAGGTGATTGAGCAACCTCAGGAAGACTTGTGGTGGATACGTCGCCTTCAGACAGCTCGAAGTCTTCCTGC
CCATGATCAGAACGAGATGGGGTTGCAATAGGCGTATCTTGACCCGTGGTGATAGGAGTATCCTGTTGAGGA
TACTCTGTAACTTGAGGACACTCTGTAACTTGAGAAGGTACTGTAGGCAGATAGACCGGAGGAGCTTCAAAT
GCAGTACCATCTTGCCAAGCTTTAAGCAAACCAGAGTTGTGTTTCACAAGCAGATGTTTATACTGTCCAGTG
AATGGGAAGGTCTGCTCATCGAAGATAACGTGGCGAGAGATGTAAACTCGACCTGTTGGTGGATGCAAACA
CCTGTAACCTTTGTACTGAGGATGATAACCGAGGAAGACACACTGTAAGGATCGGGGCTCGAACTTGTGTT
GAGCATAGGGACGCAAGCACGGATAGCACGCAGAGCCAAAGATTCTGAGAAATGAGTAGTCTGGTGCTTT

ACTAGAGAGAACCTCTGAAGGGCTTTTGTTGTTCAGAACAGAAGAAGGGATCATGTTGCTGATGAAGTTAG
CAGAGTAGAAGGCCTCAACCCAATATTTAAGAGGAGTGTGACTTTGAAATAACATAGAAAGACCCAATTCAG
TAAGATGTCTATGTTTTCTCTCAGCAATACCATTTTGTTGAGGAGTTGAAGGACATGAAACAAGATGAACAAT
CCCTTGATCTCTGAGATGATTGCGAAACCTTGTGCTCATAAACTCACCACCCCCATCGCTCTGAAAGACTTTC
AGTTTCTTGTTAAACTGATTTTCGATTTGTTTTTGAAAGACAATGAACACATCACAAAAATCAGACTTGGCTT
TAAGAGGTATTATCCATGAGTAGCGACTAAAGTTGTCAACAAAGACTGCATAATACTTGAACCCTTGAACTGA
TACAACCGGAGAAGGACCCCACAGATCACAATGTATCCGATCTATGGGTTCCTTGACACTAGACTCTGAAATA
AAAAAAGGAAGCTGACTGCTCTTTCCCATCTGGCAAGGTCCACAAACGGGAGAAATGCTGCTCTTATTGACT
ATGATTGCCTTGCTTTGCTTGAGGAGCTGAAGAATCTGGAAGTTCGCGTGCCCAAGTCGCTGATGCCACACC
ATATCACTAGCTGCACACTGACGATTGGAGAAGTAAGCCGCAAACTCCTTCTTCTCCAGCACATACAAGCCTC
TATTTCGAGTTCCCTTTGTCACCACCTTCTGAGCATCGAGGTCGATGACATAGACAGCATTAGAGTCAAAGAA
CACCCCACAGGGGTAATCATCACACAGTTTGGATACAGATAATAGTGATTTTTGCATTGAAGGACAGACAAG
AACATCGTTTAGAGGAAGGCTACCTGTCTCAGTGGTGAGCGTTGTGGAACCAACGTGGGTAATCGGAAGG
AAATTGCCATCTGCCACCATAACATGCTCAGAACCATTGTATGGAGTAGTGTTCTGCATCTGAGTTGTATTAGG
AACCATGTGAGCAGTGGCACCTGAGTCAGCTATCCATTCCCTGCCACTAGGATCAGAGACTGGAAAAGCGG
ACAGAGCATGAGGAACATCGTCACTCTGATAAGAGTTATCAAAGCGGTTCCAGCAACGAAGAGCGGAGTG
GCCGGTGCGGCCACAGATCTGACACACAGGGCGAGAGTTGTTGTTGTTATTGCCACCACCAGAGTTGCTGA
CTTGTTGAGTGAAACCTCTTCCACGAGTTGAGAAGCCACCACGACCAGAATAGCCACCGCGACCACGAGAA
TAATTATTGCTTCGTCCTCTGTTCGAGTTGTTGTAGTACCCTCGGTGAGTCTGGAAAGCCATGTTGGGAGTAA
CATCTGATGTTGCTTCATACGACTGAAGACGAGCGTCAAAACCAGAGACATCAGAAATCACATCGTTCAAAG
TCGGAGGAGGGATTCGAGTCATGGAGCTCTGAACGACAGTGATAATTGGATCATATTCTCTTCCAAGGCCAT
TGATAAATGAGAAGATCTTCATGGATTCCTCTATAGGCTTCCCAATGGAGCTCAACTTGTCACAAATGGCTCG
AAACTCACGACAGTAGGTAGTAAAGTCTTTACCTTTCTTAGTCATCAACTGGAGAGACCGTCTGAGCTCAAA
CTCACGAGCAATAGAACTCTTGTTGTAGTTGTCAGCTAACGATAACCAGACATCACGAGATGTAGATAGACCA
TGTACTGTACCAAGGACTTCCTCAGTAAGTGTTCCAAAGATCCAAGAAATGACGAGTTGATCCGTACAAGTC
CAAGCTTCAAAGCGAGGATTTGGGGCTTGAACGTTGACGTTGTCGACCACGGTTGTGACTAGCTCCGGCG
GAGGTGGAATCTGACCAGTAGCAAAGCCCAACAGCTTCTGACTGCGAAGAAGAGACTCCATTTGAGTCTTC
CATAGGAGATAGTTGCTGTCACTCAGTTTAATGGTAACAGAACTTGTAATGTGAACACTGGTGGGAAAAGG
GTATGGATCTTGCTCAGCCATGACAGCACCTGTTAGGGTTAAATGAGCTCTGATACCATGAGAACAAGAGGA
GACAGAGAGAATTTTAATAAAACTTCCTTTTGTATTCACTTTCTCAAAACGTTTCTTTACAAGATAATCGAGTA
TATATACAACCTCAAGATAAACCCTAGTTCCCTTAACTTTGGACCACTCAACACGTGTCTTCGTCACACACACT
CTACACCTTGCAAGGCATCGTAACGGCTAGACAAAGCTATTCCACAGTTTACTCCAGCTGGTGCATTTGTCTT
CTTGTTCTGTACTTGTACGAACTCTTCATTACTCTGTTTCAGAGTTTGTCTTTGGGCCAGCATACTTCCTTCAG
TTAATGGACCAGTCGCTGATATACCAAGGCCCATCATGGCTTGTTTACTGTCATCGGCGAGCTTTTGCACGGG
TCTAAAGGCGGTCATCTTCAGTGGGAGACGAGGCACAGAGTAGCCGTTGAAGCGGCGAAAGGACTGTGTT
ATCTTCACCATGACTGTTCGCCGTTGATCTTGCACAGAGACGTTAAGTCCAATAACATTCTCCTGGACTCTGAT
TTCGAAGCCCATGTTGCTGATTTTGGGCTTGCTAAGTTCTTAGTGGACGGTGCTGCTTCTGAGTGTATGTCCT
CAATAGCTGGCTCCTACGGTTACATCGCTCCAGGTTAGTTTAAACGTGTTTTATATAACAAACTATGCCATGTT
CTTTAAATGTTACTGAGACTAATCATATGTTAGAGTTTGTTGTCCGTTTTATTGTCGTATAGCTGATTACTATGAT
TTGTGACACACAAATGAATGTGACTTTAACAACTAAAATATGTACTAATCATTGTTTGTTTTGGGTTTGATAGA
GTATGCATACACTCTCAAAGTAGACGAGAAGAGTGATGTGTATAGCTTTGGAGTGGTCTTGTTGGAGCTGAT
AGCTGGGAAGAAACCGGTTGGTGAGTTTGGGGAAGGAGTGGATATAGTGAGGTGGGTGAGGAACACGGA
GGGTGAGATACCTCAGCCTTCGGATGCAGCTACTGTTGTTGCGATCGTTGACCCGAGGTTGACTGGTTACCC
GTTGACCAGTGTGATTCATGTGTTCAAGATAGCGATGATGTGTGTGGAGGATGAGGCCGCGTCAAGGCCTA

CGATGAGGGAAGTTGTGCACATGCTAACTAACTCTCCTAAGTCCGTGACTAACTTGATCGCCTTCTGAGTTCT
GACCCAATCAAAGGCTAATGAAAAAATAAGATTGTGTTGTGTGTAATGATAATATTTTTGTTTTTTTAAGTGGA
CATATATCGTTTTAAATTATTGCCGAGTTGTATGTATATTTGCTGTGTGTTGGATCTGTATATCGCCTCTTATGTG
TTTGTTATCTACTCTTAAGTCTGTAATGAGATTCTTATGCTTATTGATGATGGCTTTTAAATTTTAGCTATTTCTT
CTTTCAGACAAAGTTGCGTAAGTAATGAAAAGAGATAAAAACCAAGAAGCATATGTTCATTACAAGCAGACT
CATCATTTGTATTATCAACAAGAGCAGACAAACATGTGTCTTAGATCAAAGATGATGATGATGATCTGGAGCC
AATCCAAATGAGATCCACTCCACTTTTTCCTCTCACAAACAATTTCATCTGCTAGATTCTACCAATATCCAACTC
GAAATCTAAATATAGATTCACATGTTCATTATGGTAATAGACCCAAGATCCTCCATTACAATGTGGGATGTTCTC
TCTACACCAACAAGCCCATACAAATTACACTCAAATCCTTTAACACAGCTCTTAAATTCAAAATCATTTTTTATC
AAAAAAAAAAAAACAAGAACATTATTCATCATCGCTATAACACTAAAAAAAGAAACACAGACATAATTTTCAT
AGCTACAATAAATAACACCAATGACTAATTCCTCAATAGCTGTTATATACTTAGAAAGCACCGTAAGGTATGTG
TTGAGGAAGCGTGAATCGAACCTGCGAAGCTGGTAACCGAACCGCAACGCCGTGCCAGCAAATCCCTCCTT
TACAAATTTCACCACCTTGGTGATGCTGAAGGTGACTAAGATTCTCAACGCCTACGCGATTGTACCGCGAAA
CCGCATCCGCCGCCCTGCGAGTCACGTCCCAAGCCAGCGTGACCGCAAGCAAAACTCCGACCGTCGTAATC
GAAACTACAACGACGAATCCA

**Supplementary data 4**: cDNA length of bilocular (2964 bp including 2611 bp exon1 and 353 bp exon 2)

ATGGAGATGAGACTTTTGAAAACACACCTTCTGTTTCTGCATCTTCACTACGTTATCTCGATTTTGCTTCTATG
TTTCTCACCATGCTTCGCTTCCACTGACATGGACCATCTCCTCAACCTCAAATCCTCAATGACTGGTCACAACG
GACACGGTCTCCACGACTGGGTTCACTCCACTTCTCCCACGGCTCACTGTTCTTTCTCCGGCGTCTCATGCGA
CGCCGACGCTCGTGTTGTCTCACTCAACGTCTCTTTCACTCCTCTGTTCGGAACCATCTCACCGGAGATTGGG
ATGCTGAACCGTTTGGTGAATCTGACGTTAGCCGCGAATAACTTCTCCGGTAGGTTGCCGCTGGAGATGAAG
AGTCTCACTTCACTAAAGGTTCTCAACATCTCCAACAACGTAAACCTCAACGGAACCTTCCCCGGAGAGATT
CTCACTTCCATGGTCGACCTCGAAGTCCTCGACGCCTATAACAACAACTTCACTGGGCCGTTACCGCCGGAG
ATTCCCGGGCTGAACAAGCTCAAACACCTCTCTCTCGGAGGAAACTTCTTCACCGGAGAGATTCCCGAGAG
TTACGGAGATATCCAAAGCTTGGAGTATCTCGGCCTCAACGGAGCCGGACTCTCCGGCGAATCTCCGGCGTT
CTTGTCACGTCTCAAGAATCTCAGAGAAATGTACGTCGGCTACTTCAACAGCTACACCGGCGGCGTTCCGCC
GGAGTTCGGCGAATTAACGAAGCTAGAAATCCTCGACATGGCGAGCTGTACTCTCACCGGAGAGATTCCGA
CGACACTGAGTAATCTGAAACACTTGCACACGCTGTTCCTCCACATCAACAACTTAACCGGAAACATCCCAC
CAGAACTCTCCGGTTTAATCAGCTTAAAATCTCTCGATCTATCAATTAACCAGCTAACCGGAGAGATTCCTCA
GAGCTTCATCTCTCTGGGAAACATCACTCTCATCAACCTCTTCAGAAACAACCTCCACGGGCCGATACCGGA
GTTCATCGGAGAAATGCCGAACCTCCAAGTCTTCCAGGTGTGGGAGAACAACTTCACGTTAGAGTTACCGG
CGAATATCGGCCGGAACGGGAATCTGAAAAAGCTCGACGTCTCTGAAAACCATCTCACCGGACTCATCCCCG
TGGATTTATGCAGAGGTGGGAAGCTGGAGGTTCTGGTACTCTCCAACAACTTCTTCTTCGGCTCCATCCCGG
AGAAGCTAGGTCAATGCAAATCTCTAAACAAGATCAGAATCGTCAAGAATCTCCTCAACGGGACCGTTCCGG
CGGGACTATTCAACTTACCACTCGTTACAATCATCGAGCTCGCGGATAACTTCTTCTCCGGGGAACTTCCTAC
GGAGATGTCCGGCGACGTTCTCGATCATATCTACCTATCTAACAACTGGTTTACCGGTTTAATCCCTCCGGCTA
TCGGTAATTTCAAAAATCTACAGGATCTGTTCTTAGACCGGAACCGGTTTAGCGGGAATATTCCGAGAGAAG
TTTTCGAGCTGAAGCATCTCACGAAGATCAACACGAGCGCTAACAACCTAACCGGCGATATCCCTGACTCAT
TCTCGCGATGCACTTCCTTAATCTCCGTCGATCTCAGCCGTAACAGAATCGGCGGAGATATCCCGAAAGACAT
CCACGACGTGATTAACTTAGGAACTCTCAATCTCTCCGGGAATCAGCTCACCGGCTCGATCCCGATCGGAATC
GGGAAGATGACGAGCTTAACCACTCTCGATCTCTCCTTCAACGACCTCTCCGGGAGAGTACCACTCGGCGG
TCAGTTCCTAGTCTTCAACGACACTTCCTTCGCCGGAAACCCTTACCTCTGCCTCCCTCACCACGCCTCCTGC

CTAACGCGTCCAGGACAAACCTCTGATCGCATCCACGCGGCGCTGTTCTCTCCGTCGAGGATCGTCATCACG
ATCATCGCGGCGATCACGGCGTTGATCCTCATCAGCGTCGCGATCCGTCAGATGAACAAGAAGAAACACGA
GAGATCCCTCTCGTGGAAGCTAACCGCCTTCCAGCGACTCGATTTCAAGGCGGAAGACGTCCTCGAGTGCC
TTCAAGAAGAGAACATAATCGGCAAAGGCGGAGCGGGGATCGTCTACCGCGGATCCATGCCGAACAACGT
AGACGTCGCGATCAAACGGTTAGTTGGACGCGGAACGGGGAGGAGCGATCACGGATTCACGGCGGAGAT
TCAGACGTTAGGAAGAATCCGCCACCGTCATATAGTGAGACTACTCGGATACGTGGCGAACAAGGACACGA
ACCTGCTTCTCTACGAGTACATGCCTAACGGGAGCCTCGGCGAGCTTTTGCACGGGTCTAAAGGCGGTCATC
TTCAGTGGGAGACGAGGCACAGAGTAGCCGTTGAAGCGGCGAAAGGACTGTGTTATCTTCACCATGACTG
TTCGCCGTTGATCTTGCACAGAGACGTTAAGTCCAATAACATTCTCCTGGACTCTGATTTCGAAGCCCATGTT
GCTGATTTTGGGCTTGCTAAGTTCTTAGTGGACGGTGCTGCTTCTGAGTGTATGTCCTCAATAGCTGGCTCCT
ACGGTTACATCGCTCCAGAGTATGCATACACTCTCAAAGTAGACGAGAAGAGTGATGTGTATAGCTTTGGAG
TGGTCTTGTTGGAGCTGATAGCTGGGAAGAAACCGGTTGGTGAGTTTGGGGAAGGAGTGGATATAGTGAG
GTGGGTGAGGAACACGGAGGGTGAGATACCTCAGCCTTCGGATGCAGCTACTGTTGTTGCGATCGTTGACC
CGAGGTTGACTGGTTACCCGTTGACCAGTGTGATTCATGTGTTCAAGATAGCGATGATGTGTGTGGAGGATG
AGGCCGCGTCAAGGCCTACGATGAGGGAAGTTGTGCACATGCTAACTAACTCTCCTAAGTCCGTGACTAACT
TGATCGCCTTCTGA

**Supplementary data 5**: cDNA length of multilocular (2349 bp)
ATGGAGATGAGACTTTTGAAAACACACCTTCTGTTTCTGCATCTTCACTACGTTATCTCGATTTTGCTTCTATG
TTTCTCACCATGCTTCGCTTCCACTGACATGGACCATCTCCTCAACCTCAAATCCTCAATGACTGGTCACAACG
GACACGGTCTCCACGACTGGGTTCACTCCACTTCTCCCACGGCTCACTGTTCTTTCTCCGGCGTCTCATGCGA
CGCCGACGCTCGTGTTGTCTCACTCAACGTCTCTTTCACTCCTCTGTTCGGAACCATCTCACCGGAGATTGGG
ATGCTGAACCGTTTGGTGAATCTGACGTTAGCCGCGAATAACTTCTCCGGTAGGTTGCCGCTGGAGATGAAG
AGTCTCACTTCACTAAAGGTTCTCAACATCTCCAACAACGTAAACCTCAACGGAACCTTCCCCGGAGAGATT
CTCACTTCCATGGTCGACCTCGAAGTCCTCGACGCCTATAACAACAACTTCACTGGGCCGTTACCGCCGGAG
ATTCCCGGGCTGAACAAGCTCAAACACCTCTCTCTCGGAGGAAACTTCTTCACCGGAGAGATTCCCGAGAG
TTACGGAGATATCCAAAGCTTGGAGTATCTCGGCCTCAACGGAGCCGGACTCTCCGGCGAATCTCCGGCGTT
CTTGTCACGTCTCAAGAATCTCAGAGAAATGTACGTCGGCTACTTCAACAGCTACACCGGCGGCGTTCCGCC
GGAGTTCGGCGAATTAACGAAGCTAGAAATCCTCGACATGGCGAGCTGTACTCTCACCGGAGAGATTCCGA
CGACACTGAGTAATCTGAAACACTTGCACACGCTGTTCCTCCACATCAACAACTTAACCGGAAACATCCCAC
CAGAACTCTCCGGTTTAATCAGCTTAAAATCTCTCGATCTATCAATTAACCAGCTAACCGGAGAGATTCCTCA
GAGCTTCATCTCTCTGGGAAACATCACTCTCATCAACCTCTTCAGAAACAACCTCCACGGGCCGATACCGGA
GTTCATCGGAGAAATGCCGAACCTCCAAGTCTTCCAGGTGTGGGAGAACAACTTCACGTTAGAGTTACCGG
CGAATATCGGCCGGAACGGGAATCTGAAAAAGCTCGACGTCTCTGAAAACCATCTCACCGGACTCATCCCCG
TGGATTTATGCAGAGGTGGGAAGCTGGAGGTTCTGGTACTCTCCAACAACTTCTTCTTCGGCTCCATCCCGG
AGAAGCTAGGTCAATGCAAATCTCTAAACAAGATCAGAATCGTCAAGAATCTCCTCAACGGGACCGTTCCGG
CGGGACTATTCAACTTACCACTCGTTACAATCATCGAGCTCGCGGATAACTTCTTCTCCGGGGAACTTCCTAC
GGAGATGTCCGGCGACGTTCTCGATCATATCTACCTATCTAACAACTGGTTTACCGGTTTAATCCCTCCGGCTA
TCGGTAATTTCAAAAATCTACAGGATCTGTTCTTAGACCGGAACCGGTTTAGCGGGAATATTCCGAGAGAAG
TTTTCGAGCTGAAGCATCTCACGAAGATCAACACGAGCGCTAACAACCTAACCGGCGATATCCCTGACTCAT
TCTCGCGATGCACTTCCTTAATCTCCGTCGATCTCAGCCGTAACAGAATCGGCGGAGATATCCCGAAAGACAT
CCACGACGTGATTAACTTAGGAACTCTCAATCTCTCCGGGAATCAGCTCACCGGCTCGATCCCGATCGGAATC
GGGAAGATGACGAGCTTAACCACTCTCGATCTCTCCTTCAACGACCTCTCCGGGAGAGTACCACTCGGCGG
TCAGTTCCTAGTCTTCAACGACACTTCCTTCGCCGGAAACCCTTACCTCTGCCTCCCTCACCACGCCTCCTGC

CTAACGCGTCCAGGACAAACCTCTGATCGCATCCACGCGGCGCTGTTCTCTCCGTCGAGGATCGTCATCACG
ATCATCGCGGCGATCACGGCGTTGATCCTCATCAGCGTCGCGATCCGTCAGATGAACAAGAAGAAACACGA
GAGATCCCTCTCGTGGAAGCTAACCGCCTTCCAGCGACTCGATTTCAAGGCGGAAGACGTCCTCGAGTGCC
TTCAAGAAGAGAACATAATCGGCAAAGGCGGAGCGGGGATCGTCTACCGCGGATCCATGCCGAACAACGT
AGACGTCGCGATCAAACGGTTAGTTGGACGCGGAACGGGGAGGAGCGATCACGGATTCACGGCGGAGAT
TCAGACGTTAGGAAGAATCCGCCACCGTCATATAGTGAGACTACTCGGATACGTGGCGAACAAGGACACGA
ACCTGCTTCTCTACGAGTACATGCCTAACGGGAGCCTCGGCTGA
