# Supplementary material for: Comparative Mapping Combined With Map-Based Cloning of the Brassica juncea Genome Reveals a Candidate Gene for Multilocular Rapeseed
Source: Front Plant Sci. 2018 Nov 27;9:1744. doi: 10.3389/fpls.2018.01744 (PMC6277901; doi:10.3389/fpls.2018.01744)
Supplement: Supplementary file 2 [file Data_Sheet_2.PDF]

Supplementary figure 1 Phylogenetic tree of the *BjuB.CLV1* homologous CDSs. The neighbor joining method was used to construction phylogenetic tree by MEGA5. The names of protein were designed by the species name in NCBI. The lengths of the branches represent the amino variation rates.
