# Supplementary figures and images for: Comparative Mapping Combined With Map-Based Cloning of the Brassica juncea Genome Reveals a Candidate Gene for Multilocular Rapeseed
Source: Front Plant Sci. 2018 Nov 27;9:1744. doi: 10.3389/fpls.2018.01744 (PMC6277901; doi:10.3389/fpls.2018.01744)

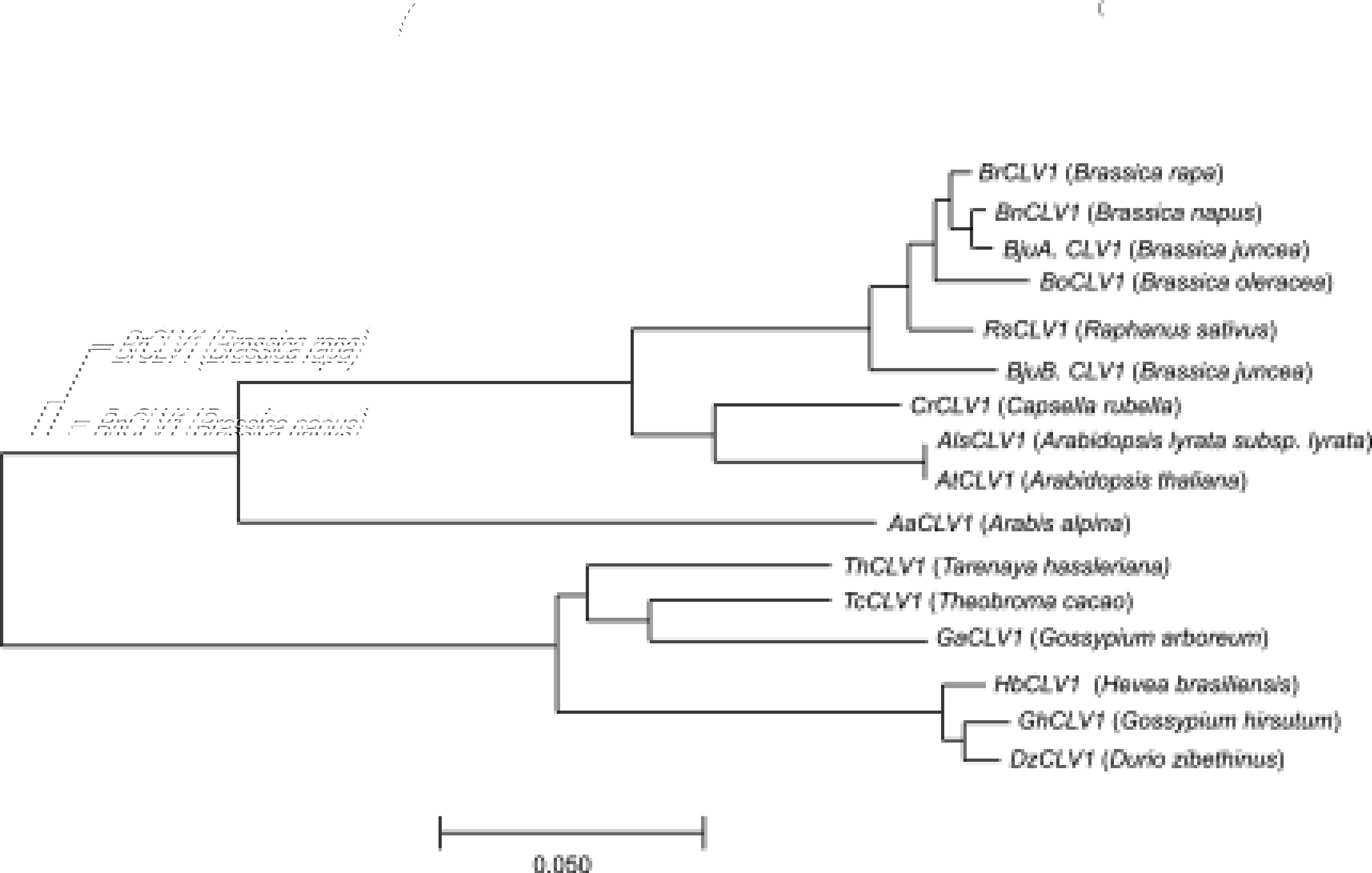

Supplement: Supplementary file 3 [file Image_1.JPEG]
